# Integrated machine learning and transcriptomics reveal immune infiltration-related orthologous transcription genes in cerebral ischemic injury

**DOI:** 10.3389/fimmu.2026.1700109

**Published:** 2026-08-11

**Authors:** Hui Xu, Xu Huang, Ming-yang Hong, Jian Xu, Tao-tao Yang, Rui-rui Shi, Jin-hua Gu

**Affiliations:** 1 Nantong Institute of Genetics and Reproductive Medicine, Department of Pharmacy, Affiliated Maternity & Child Healthcare Hospital of Nantong University, Nantong, China; 2 Nantong Key Laboratory of Genetics and Reproductive Medicine, Nantong, China; 3 Laboratory Department of the Affiliated Nantong Hospital of Shanghai University (the Sixth People’s Hospital of Nantong), Nantong, China

**Keywords:** biomarker, cerebral ischemic injury, microglia, neuroimmune dysregulation, orthologous transcription factors

## Abstract

**Background:**

Ischemic brain injury is a major contributor to global mortality and disability. Despite extensive pathological characterization, systematic integration of rodent transcriptomic data remains limited. This study investigates orthologous transcription factors (TFs) in cerebral ischemia models and their roles in regulating neuroinflammation to develop novel diagnostic and/or predictive biomarkers.

**Methods:**

We employed an integrated bioinformatics approach to analyze RNA-seq data from ten public datasets. Robustly up- and down-regulated differentially expressed genes (DEGs) were first identified from integrated rat and mouse datasets, followed by functional enrichment analysis. Orthologous TFs co-expressed in both species were screened from these robust DEGs and functionally characterized. Using machine learning, a diagnostic biomarker panel comprising five TFs (*Atf3*, *Maff*, *Cebpa*, *Myc*, and *Relb*) was identified from these conserved TFs, and the model’s clinical value and diagnostic efficacy were evaluated. CIBERSORT-based immune cell infiltration analysis was performed using public datasets and in-house samples (SD rat HIE model; C57BL/6 mouse MCAO model), revealing associations between biomarkers and macrophages. Spatial localization was further assessed via scRNA-seq data. Key findings were validated *in vitro* using qRT-PCR and immunofluorescence staining.

**Results:**

Robust DEGs common to rats and mice were primarily enriched in immune cell differentiation, immune responses, and synaptic signaling. Screening identified 51 orthologous TFs similarly enriched in leukocyte differentiation and development pathways. The machine learning-derived biomarker panel (*Atf3*, *Maff*, *Cebp*a, *Myc*, *Relb*) demonstrated high diagnostic performance. Immune infiltration analyses revealed significant associations among *Atf3*, *Cebpa*, *Relb*, and macrophages across datasets and models. scRNA-seq localized their predominant expression to microglial cells. *In vitro* validation confirmed that *Cebpa* and *Relb* are predominantly localized within microglia.

**Conclusion:**

This study identifies *Cebpa* and *Relb* as orthologous TFs in rodent cerebral ischemic injury and reveals their functional association with neuroimmune dysregulation, suggesting their potential as novel biomarkers.

## Introduction

Ischemic brain injury is a severe neurological disorder with high morbidity and disability, impairing patients’ quality of life and survival (1). Post-ischemic hypoxia triggers complex pathological mechanisms, including apoptosis, necroptosis, autophagy, and ferroptosis (2). Resultant synaptic damage causes permanent neurological deficits (3). Ischemia-reperfusion generates reactive oxygen species and activates neuroinflammation (4, 5). Current acute-phase therapies (e.g., pharmacological and interventional) show limited efficacy due to narrow therapeutic windows and restricted indications (6). Therefore, exploring novel therapeutic targets and biomarkers is critical.

Transcription factors (TFs) are proteins that bind specific DNA sequences to regulate target gene expression, and alterations in their expression influence brain damage and recovery. Orthologous TFs exhibit high cross-species sequence and regulatory network conservation, recognizing similar elements to regulate identical gene sets involved in differentiation, development, and stress responses (7). Their preservation by natural selection underscores core biological functions and their value as cross-species biomarkers for understanding pathogenesis, diagnosis, and treatment. For instance, T-bet is highly conserved in mouse and human CD4^+^ T cells, regulating consistent gene sets and offering insights into immune evolution (8). Furthermore, orthologous TFs participate in regulating longevity and represent potential anti-aging targets (9), implicating their capacity as promising biomarkers.

Microglia, the brain’s primary immune cells, maintain central nervous system homeostasis by responding to and contributing to pathologies (10). Under disease conditions, they adopt diverse transcriptionally regulated phenotypes with both beneficial and deleterious effects, including tissue support, excessive synaptic pruning, neuropathic pain, and neurodegeneration (11). IRF8 deficiency reduces microglial populations in mice (12), while PU.1, IRF8, SALL1, and SMAD4 form an enhancer complex that determines microglial identity (13). Despite extensive research, how orthologous TFs control microglial gene expression during development, homeostasis, and pathology in rodent models remains poorly understood (14). Translating these transcriptional findings from rodents to humans is essential for deeper insights into cerebral ischemia pathophysiology.

Despite significant progress in understanding ischemic brain injury mechanisms, most fundamental research remains confined to rodent models, with an extremely low clinical translation success rate (15). This profound translational gap underscores the urgent need to identify novel biomarkers and therapeutic targets. However, existing studies have largely focused on single species, single models, or single time points, lacking systematic integration of cross-species and cross-model data resources, thereby limiting the identification of core regulatory factors that are stably present across diverse biological contexts. More importantly, the conserved regulatory roles of orthologous transcription factors in neuroimmune responses following cerebral ischemia remain largely unreported, particularly their functional mechanisms in microglia—a key cell type—have yet to be elucidated. Therefore, systematically integrating multi-species and multi-model transcriptomic data, combined with machine learning and experimental validation, to screen and identify orthologous transcription factors conserved in rodent cerebral ischemia and to elucidate their mechanisms in neuroimmune regulation, not only helps to fill current research gaps but also provides a potential foundation for the development of cross-species diagnostic markers and therapeutic targets.

## Methods

### Study design

**Figure 1:**
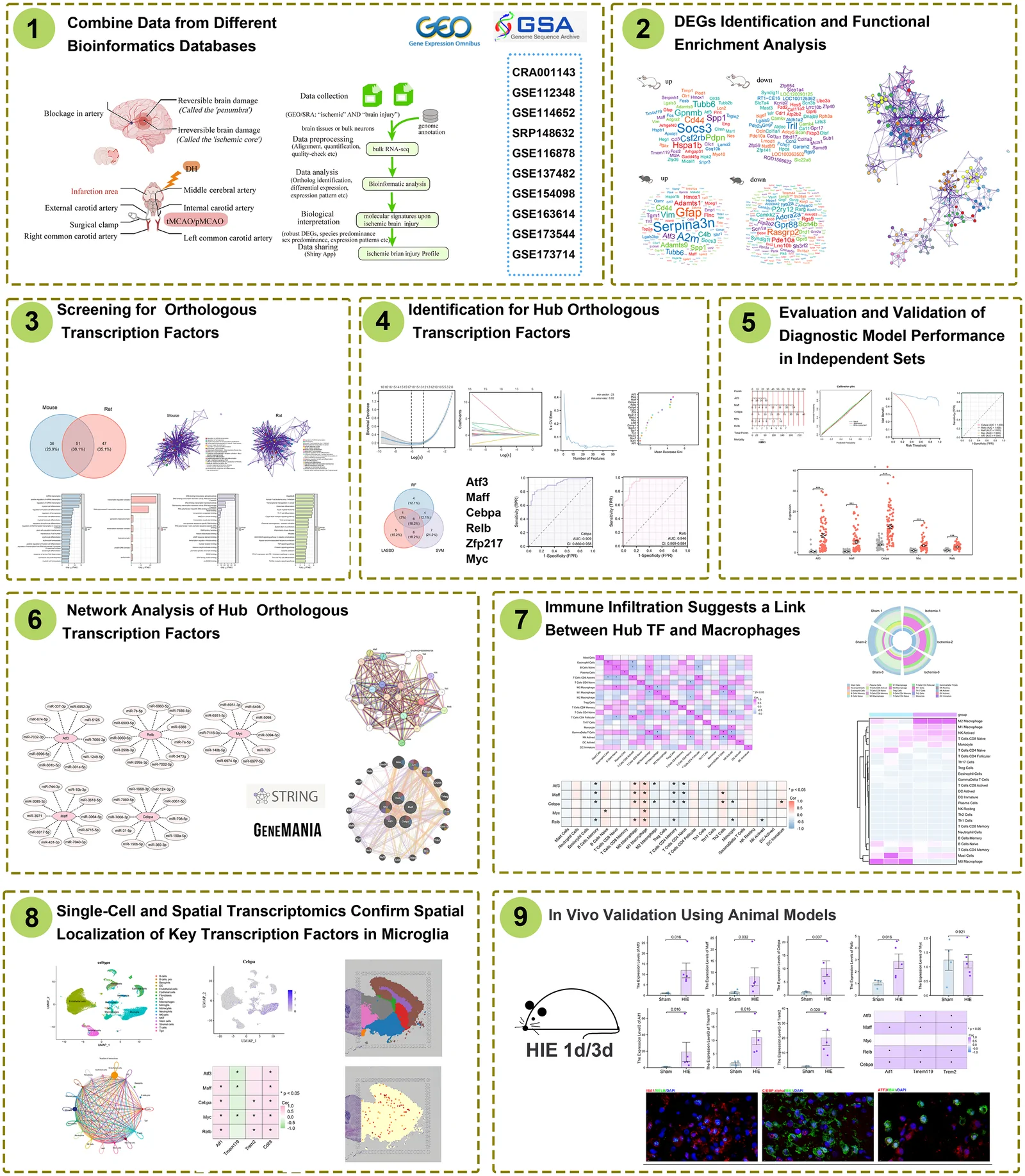
Schematic diagram of experimental design and workflow.

In this study, ten public datasets were integrated to identify robustly up- and down-regulated differentially expressed genes (DEGs), followed by functional enrichment analysis and the screening of orthologous TFs co-expressed in both mice and rats. Next, core TFs were identified using machine learning algorithms, and their diagnostic potential was evaluated, with subsequent validation using the GSE32534 dataset and in-house rodent datasets (adult mouse Middle Cerebral Artery Occlusion (MCAO) and neonatal SD rat Hypoxic-Ischemic Encephalopathy (HIE) models). A regulatory network interaction analysis was then conducted. Additionally, the association between key orthologous TFs and immune infiltration was explored using both public and in-house rodent datasets. Finally, through single-cell sequencing and experimental validation, the expression of orthologous TFs in specific cell types was localized. The experimental design and workflow are illustrated in **Figure 1**.

### Data collection

The terms “ischemic” and “brain injury” were used to search the Sequence Read Archive (SRA), Gene Expression Omnibus (GEO), and Genome Sequence Archive (GSA) databases to obtain publicly available expression profiles (last accessed in December 2021). Considering the inaccuracies in low-expressed genes, the inconsistency of array platforms, and the reliance of microarrays on prior probe design, we exclusively gathered and re-analyzed publicly available RNA-seq datasets about ischemic stroke (including permanent MCAO (pMCAO) and transient MCAO (tMCAO), and distal hypoxic) by employing universal standards. Low-quality datasets were excluded from the analysis. Each study’s sample data was meticulously reviewed by cross-referencing it with the original paper. Due to variations in research objectives across experimental paradigms, test conditions, subjects, and tissue types, the raw data were filtered using inclusion and exclusion criteria. This process resulted in the final selection of 10 datasets (**Table 1**), comprising seven studies on Mus musculus and three on Rattus norvegicus, totaling 129 samples (55 sham and 74 ischemia). Inclusion criteria: (1) Original experimental studies; (2) High-throughput sequencing data; (3) Publicly accessible raw data. Exclusion criteria: (1) non-ischemic stroke samples; (2) Duplicate datasets; (3) Gene expression microarrays. The group designation details for all utilized data are provided in **Supplementary Table 1**. SRP136487, SRP148460, SRP148632, SRP152966, SRP221729, SRP271105, SRP298712, SRP316861, and SRP318167 were obtained from the SRA database (https://www.ncbi.nlm.nih.gov/sra). CRA001143 was sourced from the GSA (https://ngdc.cncb.ac.cn). These combined external datasets were used as the training set, with GSE32529 and our in-house sequencing data constituting the test set for model evaluation. Data filtering, preparation, and batch-effect correction were performed in R (v4.2.1). Subsequent dimensionality reduction analysis was conducted using Uniform Manifold Approximation and Projection (UMAP) via the umap package (v0.2.10.0). Results from Principal Component Analysis (PCA), UMAP, and boxplot visualizations were generated using the ggplot2 package (v3.4.4).

**Table 1:** The details of the datasets.

GEO accession	SRP accession	Data type	Experiment type	Species
NA	CRA001143	Tissue	RNA-Seq, miRNA-seq	M
GSE112348	SRP136487	Tissue	RNA-Seq	M
GSE114652	SRP148460	Tissue	RNA-Seq	M
NA	SRP148632	Tissue	RNA-Seq	R
GSE116878	SRP152966	Tissue	RNA-Seq	M
GSE137482	SRP221729	Tissue	RNA-Seq	M
GSE154098	SRP271105	Tissue	RNA-Seq	R
GSE163614	SRP298712	Tissue	RNA-Seq	R
GSE173544	SRP316861	Tissue	RNA-Seq	M
GSE173714	SRP318167	Tissue	RNA-Seq	M
GSE174574	SRP320164	Tissue	scRNA-seq	M
GSE158450	SRP285126	Tissue	10x Visium, scRNA-seq	M
GSE32529	NA	Tissue	Microarray	M

### Identification of orthologous DEGs upon injury

Differential expression analysis was performed using the Limma package (v3.40.6). Genes exhibiting a |log2 ^fold-change^| ≥ log2^1.5^ with an adjusted *P*-value<0.05 were identified as significant DEGs. To identify robust injury-related genes, we selected genes present in most of the compared groups in each injury model involving rats or mice (e.g., 50%). The score for each differentially expressed gene was computed in accordance with the subsequent regulations:


$$x_{ij}=\;\{\begin{array}{c} 1\;\left(up\right)\\ -1\;\left(down\right) \end{array}\;;\;score_{i}=\sum_{\left(j=1\right)}^{N}x_{i}j\;$$

where N is the total number of compared groups.

Differentially expressed genes with a score≥50%×N were defined as orthologous differentially expressed genes (16).

### Identification of the orthologous relationship of protein-coding genes between mouse and rat

The protein sequences were initially obtained from the Gencode (mouse: vM27) and NCBI (rat: mRatBN7.2) databases. Representative sequences for the gene were determined by selecting the most extended protein sequences from genes that contain multiple isoforms. Except for BLAST, which was used as an aligner for comparative analysis, orthologous relationships between mice and rats were identified using the OrthoFinder pipeline with default parameters (17). Genes that shared a single-copy orthologous relationship or had the same gene nomenclature were chosen to compare the responses of rats and mice to ischemic stroke.

### Functional enrichment analysis

Gene Ontology (GO) annotation and Kyoto Encyclopedia of Genes and Genomes (KEGG) pathway enrichment analyses of stably up-or down- regulated differentially expressed genes were conducted separately using the clusterProfiler package (v4.0.5). In addition, the Metascape tool (18) (http://metascape.org/) was utilized to perform GO and KEGG enrichment analyses. Orthologous differentially expressed TFs were input, and species-specific parameters were selected to generate visualization outputs. This approach elucidated the functional roles of pathways associated with the screened candidate TF genes.

### Selection of machine learning algorithms and identification of orthologous hub TFs in rodent species

This study employed three machine learning algorithms—Least Absolute Shrinkage and Selection Operator (LASSO), Random Forest (RF), and Support Vector Machine-Recursive Feature Elimination (SVM-RFE)—via an online analysis platform (https://www.xiantaozi.com/) to identify hub TFs. Specifically, the glmnet package (v4.1.7) in R was used to perform LASSO regression analysis with 10-fold cross-validation and the random seed set to 2022; the randomForest package (v4.7.1.1) was applied to construct a Random Forest model with 100 decision trees and the random seed set to 2024; and the e1071 package (v1.7.13) was utilized to build the Support Vector Machine model using 5-fold cross-validation with the random seed set to 2024. Finally, the intersection of results from these methods was identified as hub TFs associated with ischemic stroke. To evaluate their diagnostic accuracy, Receiver Operating Characteristic (ROC) curves were generated using the pROC package (v1.18.0) to assess the efficacy of the identified key feature genes. An area under the curve (AUC) value > 0.7 was considered indicative of acceptable diagnostic performance.

### Nomogram and decision curve analysis

The rms package (v6.4.0) was employed to construct a nomogram model using the five identified signature TFs. Based on their association with ischemic stroke, multivariate logistic regression was used to assign regression coefficients to each hub gene. These coefficients were subsequently used to compute a risk score for each gene. Calibration curves were subsequently plotted using the rms package to visualize agreement between predicted and actual probabilities and to evaluate model performance. The rmda package (v1.6) was employed to compute and visualize Decision Curve Analysis curves. Decision Curve Analysis demonstrates the variation in net benefit values when implementing interventions based on model predictions across different threshold probabilities, a widely used approach to assess the clinical utility of predictive models.

### Network interaction analysis

Network interaction analysis was performed using STRING (https://cn.string-db.org/) and GeneMANIA (https://genemania.org/) to predict interactions between key TFs and proteins. The TargetScan website (https://www.targetscan.org/) was employed to identify miRNAs predicted to regulate orthologous TFs. Subsequently, significant networks were constructed and visualized using Cytoscape software (v3.9.1).

### Homologous gene conversion

Orthologous gene conversion between mouse and rat was performed using the Homologene package (v1.4.68.19.3.27).

### Assessment of the immune landscape

The proportions of various immune cell types within samples can be calculated using the CIBERSORT algorithm on the website (https://www.bioinformatics.com.cn). We used this algorithm to analyze the immune cells in CRA001143, SRP221729, SRP148632, and our self-generated mouse and rat datasets. Subsequently, Spearman or Pearson correlation analysis was then performed to evaluate the correlations between the screened orthologous key TFs and macrophage markers or immune cells, as well as correlations among immune cells. Then visualize the results using the ComplexHeatmap package (v2.13.1).

### Single-cell RNA-sequencing data analysis

Single-cell RNA-sequencing data were analyzed using the HiOmics Cloud Platform (https://henbio.com/en/tools) (19). Briefly, the GSE174574 dataset containing single-cell transcriptome sequencing data was downloaded. This dataset comprises three samples from a mouse MCAO model of ischemic stroke and three sham control samples. Raw scRNA-seq data were processed into a Seurat object using Seurat (v5.3.0). Cells were filtered by a gene count threshold of 4,500 and a mitochondrial gene percentage threshold of 10%. Data were normalized, and highly variable genes were identified. Dimensionality reduction was performed using UMAP. The SingleR package (v3.21) was employed to annotate distinct cell subtypes and determine the proportions of different cell types within the sequenced dataset. CellChat (v2) was utilized to assess cell-cell communication between the identified cell clusters in silico.

### Spatial transcriptomics technology

To comprehensively validate the co-localization of key TFs with target cells, we utilized spatial transcriptomic data from the Brain Region- and Cell Type-Specific Isoform Atlas of the Postnatal Mouse Brain (7 days postnatal; Sample ID: STSP0003575; GEO Accession: GSE158450 (20)), available in the Spatial Transcript Omics DataBase (21) (STOMICS DB, https://db.cngb.org/stomics/). This dataset enabled evaluation of the co-localization patterns of *Atf3*, *Maff*, *Cebpa*, *Myc*, and *Relb* with microglia.

### Animals

Male C57BL/6 mice weighing 25–28 g were purchased from Vital River (Beijing, China). These mice were housed in six cages at the Laboratory Animal Center of Nantong University under standard conditions (12-hour light/dark cycle). Seven-day-old SD pups, weighing 15–16 g, were obtained from the Laboratory Animal Center of Nantong University. The pups were allowed to nurse from the mother both before and during the experimental recovery period. All animal experiments were reviewed and approved by the Animal Care Committee of Nantong University under approval number S20230813-001. The experimental procedures were conducted in accordance with the guidelines set forth by the National Institutes of Health’s Guide for the Care and Use of Laboratory Animals.

### MCAO model

Mice were placed under deep isoflurane anaesthesia (5% induction, 3.5% maintenance; RWD, Shenzhen, China). An intraluminal nylon monofilament was introduced via the external carotid artery stump and advanced 11 mm beyond the carotid bifurcation into the internal carotid artery to induce middle cerebral artery occlusion. Continuous cerebral blood flow monitoring was performed throughout the procedure using laser speckle contrast imaging (RFLSI III, RWD). Subjects failing to achieve sufficient CBF reduction (>70% baseline) or exhibiting premature reperfusion during occlusion were excluded. Following 60 min of sustained occlusion, the filament was withdrawn to initiate reperfusion, which was maintained for 24 h. The body temperature of the mice was maintained between 36.5 and 37.5 °C during the procedure using a heat lamp. Mice in the sham-operated group underwent identical procedures, except for the insertion of the filament.

### HIE model

Seven-day-old, sex-unmatched SD rats (weighing 15–16 g) were anesthetized using isoflurane (induction at 3-4%, maintenance at 1-2%). A small incision was made in the center of the neck, and the right CCA was ligated with 7–0 silk sutures using a double occlusion technique, followed by cutting with microscissors. The pups were then returned to the mother for 1 h of recovery and nursing. Subsequently, the pups were placed in a hypoxic chamber containing 8% oxygen (balance with 92% nitrogen) for 2 h, maintained at 37 °C. At designated time points, both adult and pup rats were anaesthetized with isoflurane, and brain tissue was collected. Part of the tissue was stored in 4% paraformaldehyde for 24 h before being embedded in paraffin for sectioning. Additional brain tissue was immediately frozen in liquid nitrogen for RNA extraction, while another portion was used for RNA sequencing.

### Immunofluorescence staining

For paraffin-embedded tissue sections, deparaffinized and rehydrated sections were subjected to epitope retrieval by boiling in glycine buffer (100 mM, pH 9.0) for 10 min. Sections were then blocked with PBS containing 5% goat anti-mouse IgG serum (Servicebio, Shanghai, China) at room temperature for 2 h. Sections were incubated overnight at 4 °C with diluted primary antibodies (**Supplementary Table 2**). After washing with PBS, sections were incubated with a secondary antibody for 1 h at room temperature. Nuclei were counterstained with DAPI (Servicebio), and fluorescent images were captured using a confocal laser scanning microscope (Nikon, NY, USA).

### Quantitative real-time reverse transcription polymerase chain reaction

Total RNA was extracted from brain tissue using the RNA-easy Isolation Reagent (Vazyme, Nanjing, China) and subsequently reverse-transcribed with the HiScript III RT SuperMix (Vazyme) according to the manufacturer’s protocol. qRT-PCR was performed utilizing the AceQ qPCR SYBR^®^ Green Master Mix (Vazyme) on the LightCycler 96 system (Roche, Shanghai, China). Relative gene expression was quantified using the 2⁻^ΔΔCt^ method, with *Gapdh* as the endogenous reference gene. All primer sequences are listed in **Supplementary Table 3**.

### Statistical analysis

Statistical analyses were conducted using the online platform at [https://www.xiantaozi.com/], and data are presented as mean ± standard error of the mean (SEM). For comparisons between two independent groups, the software automatically selected the appropriate test based on normality (Shapiro–Wilk) and homogeneity of variance (Levene’s) assessments: the unpaired Student’s t-test was applied when both assumptions were satisfied; Welch’s t-test was used when normality held but variances were unequal; and the Mann–Whitney U test (Wilcoxon rank-sum test) was employed when normality was violated. A P-value< 0.05 was considered statistically significant.

## Results

### Overview of public bulk RNA-seq of the brain under distinct ischemic injury

**Figure 2:**
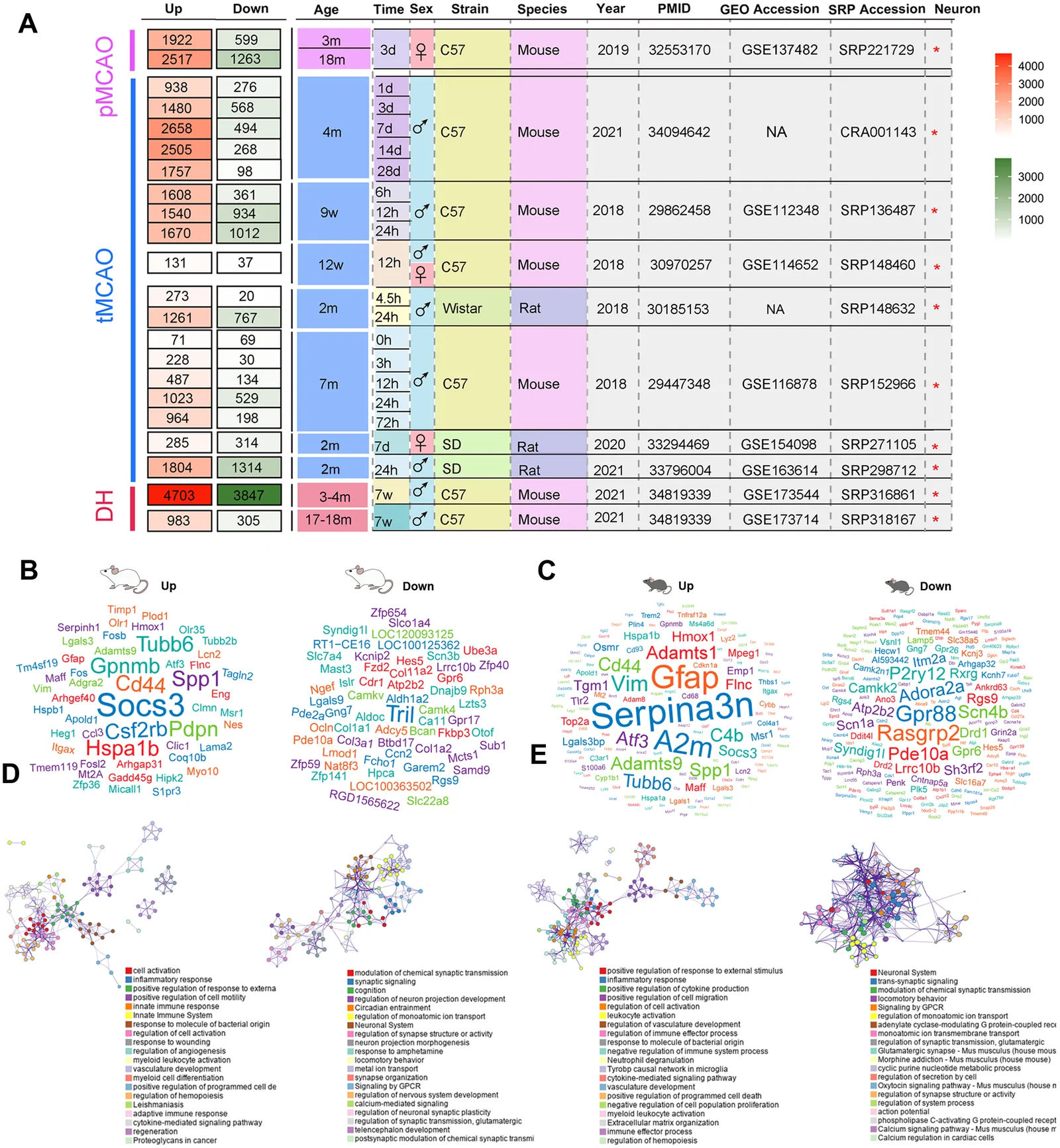
Profiling and integrating ischemic stroke datasets. **(A)** Integrated and analyzed datasets from rat and mouse stroke models. **(B, C)** Word clouds showing stably up- or down-regulated DEGs in rats and mic. **(D, E)** Pathway enrichment analysis of stably up- or down-regulated DEGs using Metascape. DEGs, Differentially expressed genes.

After searching the SRA and GSA databases and further excluding datasets with missing information, duplicate samples, merged samples, poor quality control, and no clear distinction between stroke and non-stroke groups, 10 datasets were finalized, with injury time points ranging from 3 hours to 7 weeks. Only libraries associated with injury or control were retained for each dataset. Next, we performed a uniform pipeline reanalysis, including quality control, reference-based genomic localization, gene expression quantification, and differential expression analysis (**Figure 2A**; **Supplementary Figure 1**). tMCAO was the most widely used rodent model, with 7 datasets across 2 species (rats and mice). 1 dataset and 2 datasets with the pMCAO model (mouse) and the distal hypoxic model (mouse), respectively. Different models exhibited varying degrees of molecular change, as determined by differential expression analyses using different numbers of genes (**Figure 2A**) (22).

### Functional enrichment analysis of robust differentially expressed genes

According to previous literature reports, genes scoring in the top 50% were defined as robustly DEGs (16). The Heatmap and WordCloud show the robust DEGs upon injury in rats and mice (**Supplementary Figure 2**; **Figures 2B, C**). Functional enrichment analysis of these genes was performed using the Metascape tool (**Figures 2D, E**). In rats, the stably upregulated DEGs were involved in regulating immune cell differentiation and immune responses. For the downregulated DEGs, they are primarily involved in synaptic signaling transmission within the nervous system (**Figure 2D**). In mice, the upregulated DEGs predominantly participated in immune responses, while the downregulated DEGs also show significant enrichment for functions related to neuronal synaptic signaling (**Figure 2E**).

### Identification and functional enrichment analysis of key orthologous TF in rodents

**Figure 3:**
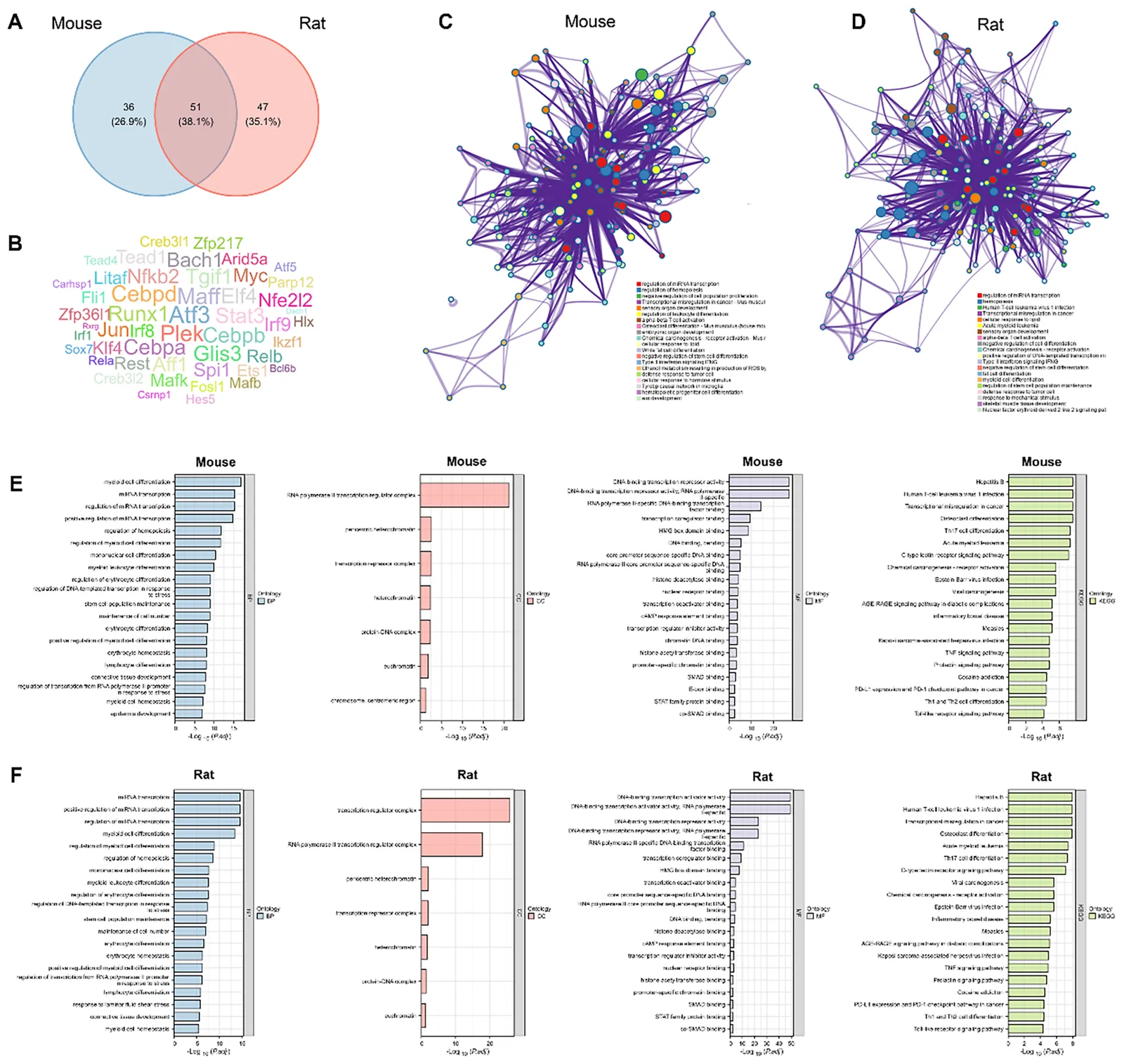
Screening and functional enrichment of 51 differential TFs. **(A, B)** Venn diagram and word cloud showing key orthologous TFs derived from stably up- or down-regulated DEGs. **(C, D)** Enrichment analysis of the 51 differentially expressed TFs using Metascape. **(E, F)** Bar graphs of GO and KEGG enrichment analyses of 51 TFs in mouse and rat. TFs, Transcription factors; GO, Gene Ontology; KEGG, Kyoto Encyclopedia of Genes and Genomes. DEGs, Differentially expressed genes.

To identify orthologous TFs, we first screened the DEGs from mouse and rat models for TFs and then determined their intersection. This analysis yielded a core set of 51 orthologous TFs (**Figures 3A, B**; **Supplementary Table 4**). Pathway and Process Enrichment Analysis in Metascape revealed that these 51 TFs were associated with the differentiation of various cell types, including stem cells, leukocytes, and osteoclasts (**Figures 3C, D**). Additionally, we performed GO and KEGG enrichment analyses. **Figures 3E, F** demonstrate significant enrichment of these TFs in: (a) Biological Processes: myeloid/mononuclear cell differentiation, regulation of erythrocyte differentiation, stress-responsive DNA-templated transcription via RNA polymerase II promoters, and myeloid cell homeostasis; (b) Cellular Components: RNA polymerase II transcription regulator and repressor complexes; (c) Molecular Functions: DNA-binding transcription repressor activity, transcription coregulator binding, histone deacetylase binding, and nuclear receptor binding; (d) KEGG Pathways: Human T-cell leukemia virus 1 infection, osteoclast differentiation, Th17/Th1/Th2 cell differentiation, C-type lectin receptor/Toll-like receptor signaling pathways, inflammatory bowel disease, and TNF signaling pathway. In summary, our results demonstrate that both the identified robustly differentially expressed genes and the orthologous TFs expressed in both mouse and rat were associated with leukocyte differentiation and development in immune cells.

### Identification of key orthologous TFs in rodents

**Figure 4:**
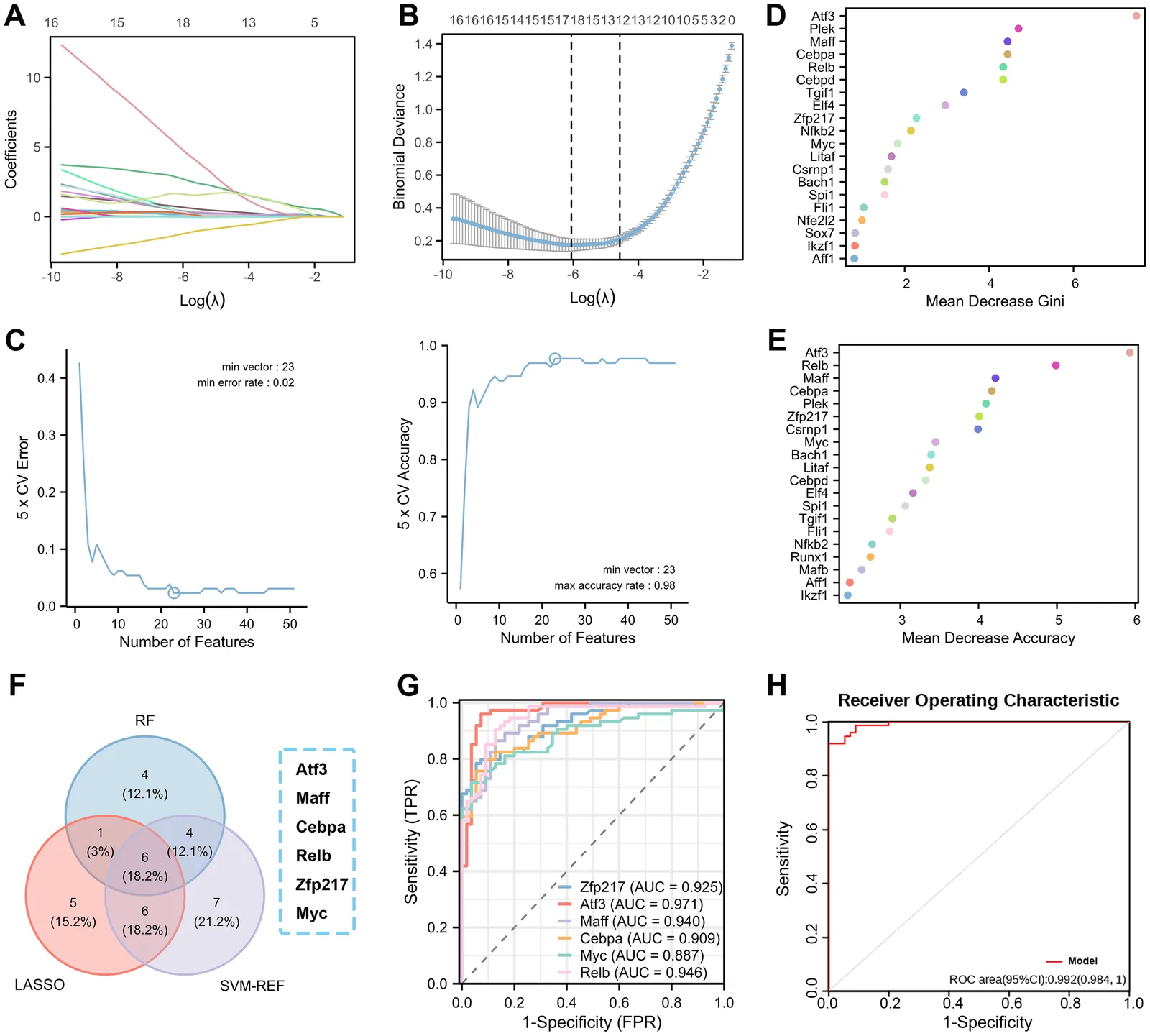
Screening hub orthologous TFs in the merged dataset by machine learning**. (A, B)** LASSO logistic regression algorithm to screen diagnostic markers. **(C)** The error and accuracy of the estimate generation for the SVM-RFE algorithm. **(D, E)** Characteristic genes identified by RF screening and their importance scores. **(F)** The overlap of genes from the three algorithms. **(G, H)** ROC curves for estimating the diagnostic performance of characteristic genes, with 95% confidence intervals (CIs) as follows: *Atf3*: 0.942-0.999; *Maff*: 0.903-0.977; *Cebp*a: 0.860-0.958; *Myc*: 0.829-0.945; *Relb*: 0.909-0.984, and *Zfp217*: 0.883-0.967). TFs, Transcription factors; LASSO, Least Absolute Shrinkage and Selection Operator. SVM-RFE, Support Vector Machine-Recursive Feature Elimination; RF, Random Forest. ROC, Receiver operating characteristic.

To further screen for conserved damage-associated TFs in rodents, we employed three commonly used feature selection algorithms: LASSO, SVM-RFE, and RF. First, LASSO regression analysis identified 18 key TF genes closely associated with diagnosis, including *Atf3*, *Maff*, *Cebpb*, *Cebpa*, *Glis3*, *Nfkb2*, *Aff1*, *Myc*, *Relb*, *Klf4*, *Mafk*, *Irf8*, *Fli1*, *Zfp217*, *Tead4*, *Bcl6b*, *Rxrg*, and *Dach1* (**Figures 4A, B**). Second, the SVM-RFE results indicated that the diagnostic model achieved optimal accuracy (0.98) with minimal error when incorporating 23 TF genes (**Figure 4C**; **Table 2**). Further analysis using Random Forest identified 15 markers significantly associated with ischemia diagnosis (importance score > 2) (**Figures 4D, E**; **Table 2**). Then, to identify common key genes for the diagnostic model, we utilized a Venn diagram, which revealed six overlapping genes: *Atf3*, *Maff*, *Cebp*a, *Myc*, *Relb*, and *Zfp217* (**Figure 4F**; **Table 2**). Finally, the ROC curves and combined diagnostic analysis for these six key genes in the merged dataset demonstrated their robust diagnostic efficacy for cerebral ischemia (**Figures 4G, H**).

**Table 2:** Key Orthologous TFs.

Machine Learning	Transcription Factors
LASSO	*Atf3*, *Maff*, *Cebpb*, *Cebpa*, *Glis3*, *Nfkb2*, *Aff1*, *Myc*, *Relb*, *Klf4*, *Mafk*, *Irf8*, *Fli1*, *Zfp217*, *Tead4*, *Bcl6b*, *Rxrg*, *Dach1*.
SVM-RF	*Plek*, *Zfp217*, *Atf3*, *Relb*, *Ikzf1*, *Aff1*, *Fli1*, *Cebpa*, *Mafk*, *Irf8*, *Elf4*, *Jun*, *Bach1*, *Spi1*, *Runx1*, *Nfe2l2*, *Maff*, *Parp12*, *Myc*, *Ets1*, *Mafb*, *Rxrg*, *Dach1*.
RF	*Atf3*, *Plek*, *Maff*, *Cebpa*, *Relb*, *Cebpd*, *Tgif1*, *Elf4*, *Zfp217*, *Nfkb2*, *My*c, *Litaf*, *Csrnp1*, *Bach1*, *Spi1*.

### The diagnostic significance and expression levels of critical TF genes

**Figure 5:**
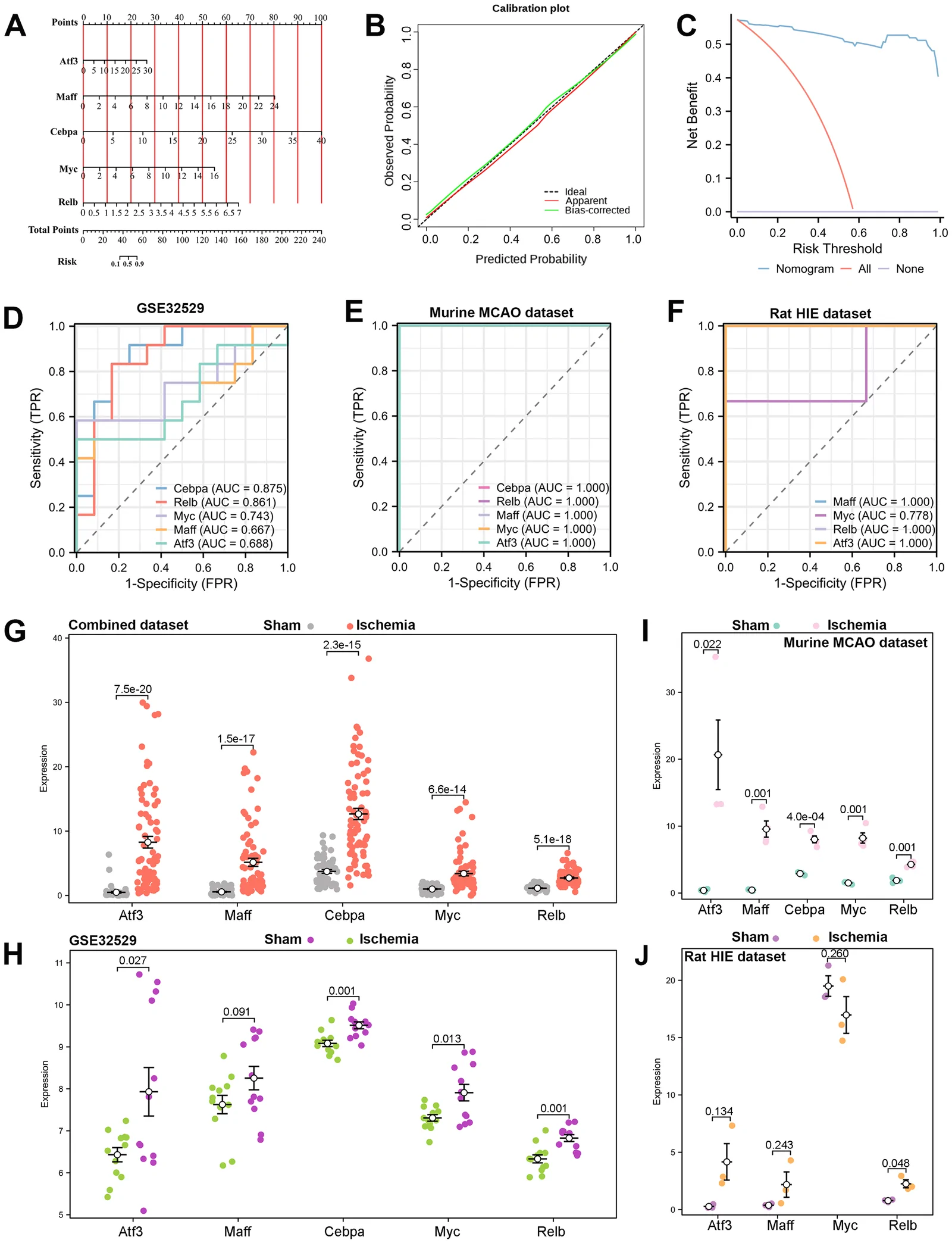
Validation of predictive ability of the model. **(A)** Nomogram for predicting ischemic stroke risk using hub TFs. **(B)** Calibration curve of the nomogram. **(C)** The decision curve analysis of the nomogram. **(D)** ROC curves from the external dataset GSE32529 (Sham, n=12; Ischemia, n=12), with 95% CIs as follows: *Atf3*: 0.459-0.916; *Maff*: 0.436-0.898; *Cebp*a: 0.727-1.000; *Myc*: 0.530-0.956; *Relb*: 0.909-0.984). **(E, F)** ROC curves in the self-test dataset from mouse and rat samples (Sham, n=3; Ischemia, n=3~4), with 95% CIs as follows: AUC = 1 (95% CI: 1.000), *Myc*:0.291-1.000). **(G)** The FPKM value of hub orthologous TFs in the merged dataset, with 95% CIs as follows: *Atf3*: 3.2119-7.311; *Maff*: 1.4611-3.872; *Cebp*a: 5.7301-9.425; *Myc*: 1.1031-1.8839; *Relb*: 1.093-1.6449). **(H)** The FPKM value of hub orthologous TFs in GSE32529, with 95% CIs as follows: *Atf3*: 0.19765-2.8029; *Maff*: -0.10887-1.368; *Cebp*a: 0.19893-0.66411; *Myc*: 0.1488-1.0547; *Relb*: 0.23495-0.75585). **(I)** Gene expression values (FPKM) for hub orthologous TFs in the self-test dataset from mouse, with 95% CIs as follows: *Atf3*: 4.479-36.058; *Maff*: 5.428-12.799; *Cebp*a: 3.5327-6.6756; *Myc*: 4.3675-9.0542; *Relb*: 1.5919-3.2097). **(J)** Gene expression values (FPKM) for hub orthologous TFs in the self-test dataset from rat, with 95% CIs as follows: *Atf3*: -2.9154-10.695; *Maff*: -2.9045-6.5158; *Myc*: -8.2246-3.1826; *Relb*: 0.037699-2.905). TFs, Transcription factors; ROC, Receiver operating characteristic. FPKM, Fragments per kilobase of exon model per million mapped fragments.

Leveraging the six diagnostic biomarkers, we developed a composite prediction tool comprising: 1) a nomogram for visual risk stratification, and 2) a calibration curve assessing model performance (**Figures 5A, B**). Five orthologous TFs—*Atf3*, *Maff*, *Cebpa*, *Myc*, and *Relb*—were incorporated into a multivariable logistic regression framework. In contrast, the *Zfp217* gene was excluded from subsequent analyses because it was completely separated in the data, preventing model convergence. Within the nomogram, scaled point assignments for each factor correspond to regression coefficients, enabling clinical risk quantification from transcriptional profiles. Calibration plots confirmed the predictive accuracy and reliability of the nomogram model. The close agreement between the calibration curve and the ideal line indicates excellent concordance between observed and predicted outcomes (C-index=0.992; Hosmer-Lemeshow goodness-of-fit test, *P* = 0.997). Decision curve analysis demonstrated substantial clinical net benefit across relevant threshold probabilities (**Figure 5C**).

To systematically evaluate the model’s diagnostic capability, we first used the published independent dataset GSE32529 for validation. In the GSE32529 dataset, *Cebpa*, *Myc*, and *Relb* maintained favorable diagnostic performance (AUC > 0.7). In contrast, the diagnostic efficacy of *Atf3* and *Maff* significantly declined (AUC< 0.7) (**Figure 5D**). Additionally, in the test set of the internal adult mouse MCAO model, five TFs demonstrated high diagnostic performance (AUC = 1) (**Figure 5D**). Furthermore, we collected data from an internal neonatal rat HIE model, which was designated as an extension study (**Figure 5F**). In this extension study, *Atf3*, *Maff*, and *Relb* exhibited strong diagnostic efficacy (AUC = 1). At the same time, *Myc* showed relatively lower diagnostic capability (AUC = 0.778). It is important to note that these internal datasets may be subject to overfitting risks due to limited sample sizes, potentially leading to biased performance evaluations. Therefore, these results should be regarded as preliminary, indicating the model’s potential efficacy, and require comprehensive analysis integrating findings across all three test sets. Our study revealed that although specific TFs, such as *Atf3* and *Maff*, performed well in internal datasets, their diagnostic validity was not corroborated in an independent published dataset. Given the relatively stable diagnostic value of *Cebpa*, *Relb*, and *Myc* across different datasets, we hypothesize that they may play more critical roles in hypoxic-ischemic brain injury.

As shown in **Figures 5G–I**, the expression levels of these characteristic TFs were significantly increased in the ischemia group within the combined dataset, and GSE32529, and similar results were observed in the murine MCAO dataset (*P* < 0.05, *P* < 0.01, and *P* < 0.001). Notably, *Relb* exhibited progressively stronger differential expressions in rat HIE samples compared to Sham controls (*P* < 0.05) (**Figure 5J**). **Supplementary Figure 3** demonstrates high consistency in the in-house dataset after normalization and batch-effect removal.

### Network interaction analysis

**Figure 6:**
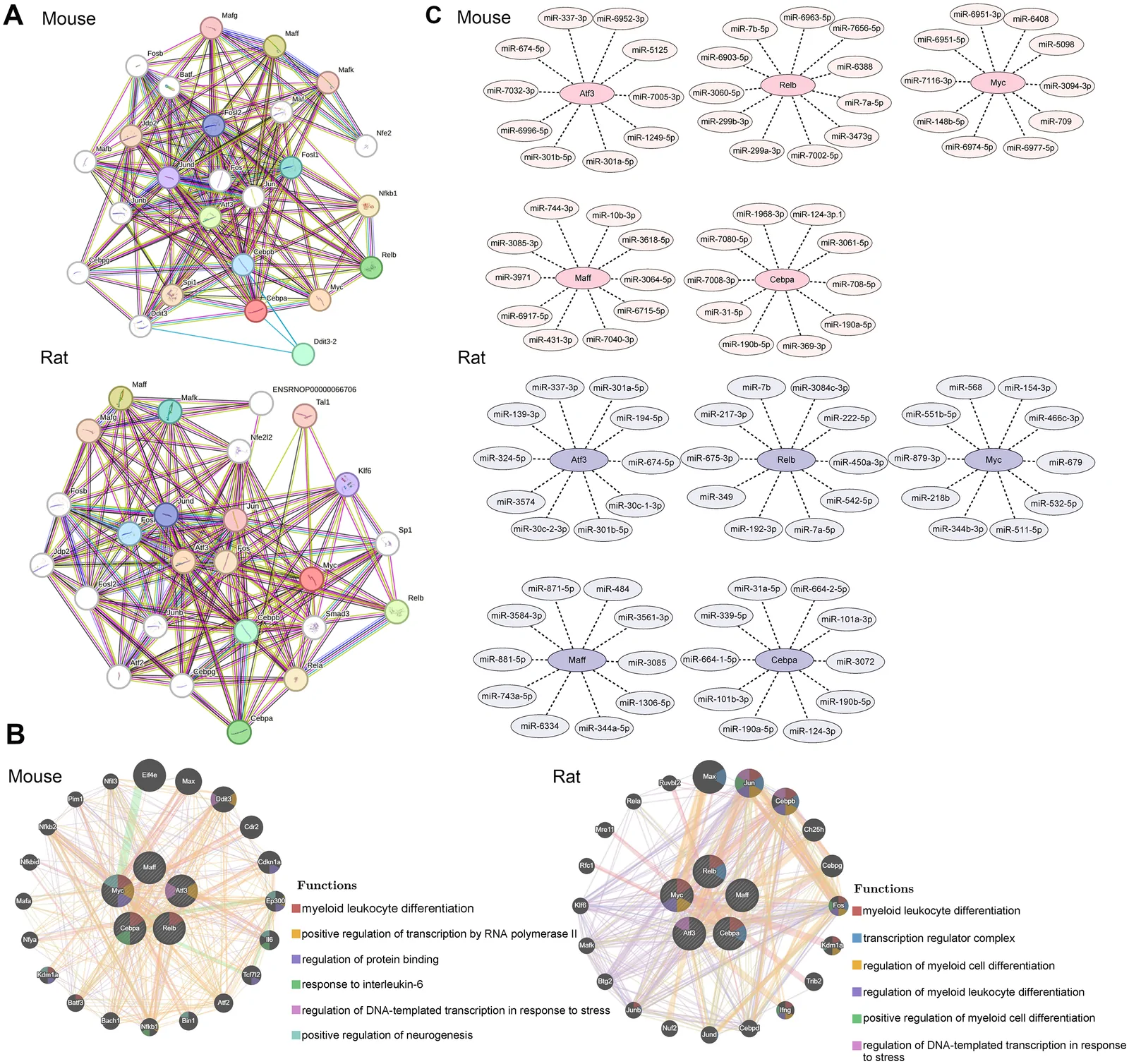
Construction of a regulatory network for key orthologous TFs. **(A)** Construction of the PPI network for key TFs using the STRING database. **(B)** TF–protein interaction network analysis of 5 differentially expressed TFs using GeneMANIA. **(C)** Predicted miRNA binding sites on TFs identified by TargetScan. TFs, Transcription factors. PPI, Protein-Protein Interaction; miRNA, microRNA.

To understand the overall framework and regulatory relationships in gene regulation, we first used the STRING and GeneMANIA databases. These tools helped predict proteins linked to five key orthologous TFs and their interactions (**Figures 6A, B**). Core TFs regulate the differentiation and activation of myeloid cells. They regulate the transcription of DNA templates under stress conditions and are also implicated in the transcriptional regulation by RNA polymerase II. We also used the TargetScan database to predict potential regulatory miRNAs for these key TFs (**Figure 6C**), providing a basis for further elucidation of the gene regulatory network.

### Correlation between key orthologous TFs and immune infiltrating cells in mice

**Figure 7:**
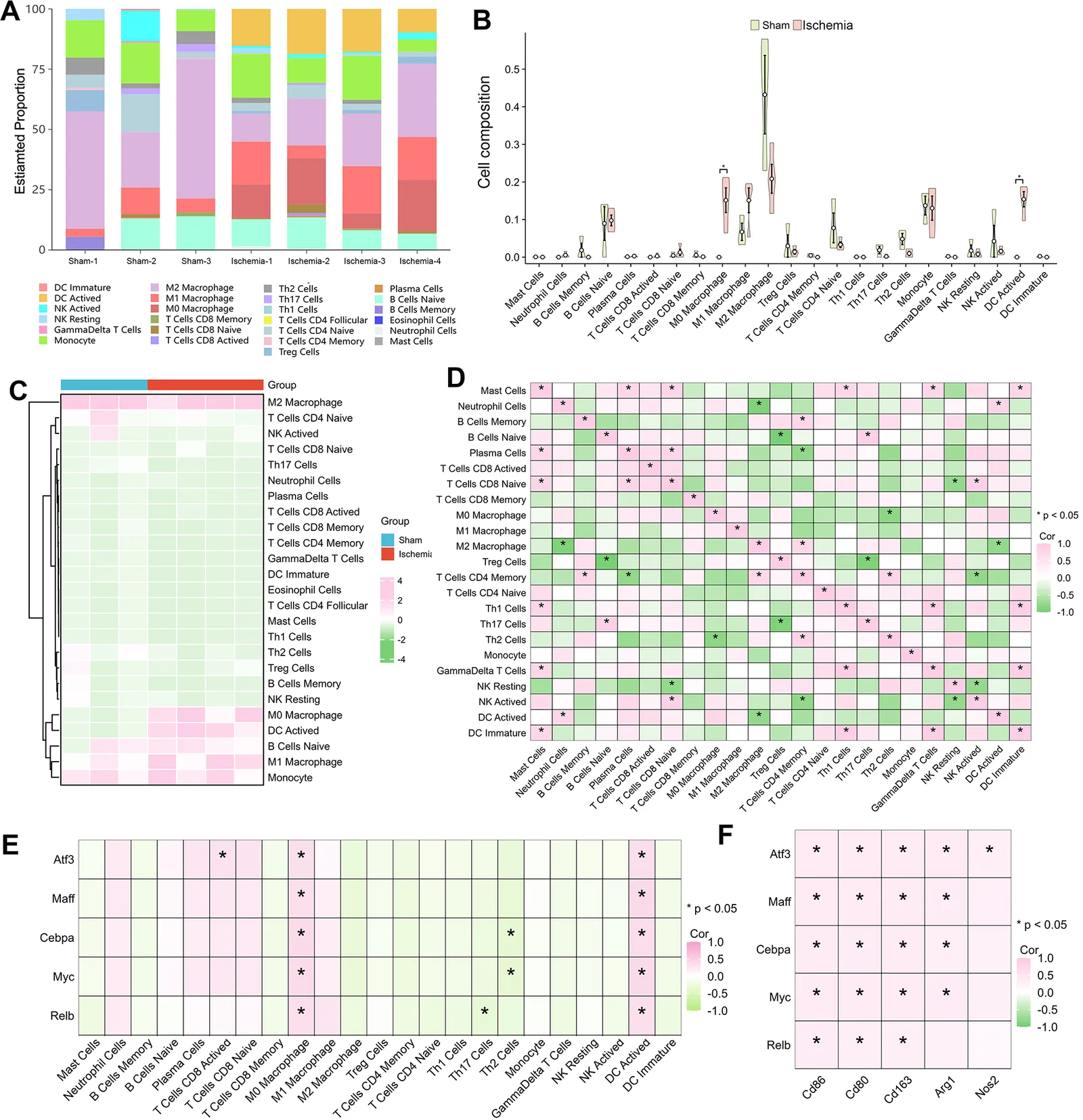
Analysis of immune cell infiltration using our self-generated mouse dataset. **(A)** Stacked histograms depict immune cell infiltration across different groups. **(B)** Comparisons of immune cell infiltration levels between the Sham and Ischemia groups. **(C)** Clustering of immune cell infiltration. **(D)** Intercellular correlations of immune cells. **(E)** Correlation between 5 hub orthologous TFs and immune cells. **(F)** Correlation analysis of the expression of 5 key TFs with M1 and M2 macrophage markers. TFs, Transcription factors. Arg1, Arginase 1. NOS2, Nitric oxide synthase 2. * *P<*0.05.

Previous results demonstrated that both the identified robustly differentially expressed genes and the orthologous TFs expressed in both rats and mice were associated with leukocyte differentiation and development in immune cells. To further validate this, we performed immune infiltration analysis using the CIBERSORT algorithm via deconvolution. **Figure 7** shows the immune cell infiltration results for the Ischemia and Sham groups in mouse self-testing. Macrophages (M0, M1, M2) constitute the predominant component of the immune cell composition in both groups, as visualized in the stacked bar chart (**Figure 7A**). Comparing the abundance of immune cells between the two groups, we found that the Ischemia group had significantly higher levels of M0 macrophages and activated DCs compared to the Sham group (*P* < 0.05; **Figures 7B, C**). A comprehensive analysis of the correlations among immune cells revealed both positive and negative interactions among various immune cells, notably between macrophages and neutrophils, diverse T cell subsets, activated dendritic cells (**Figure 7D**). Further investigations revealed that the expression levels of 5 key orthologous TF genes in the self-generated dataset were positively correlated with M0 macrophages and activated DCs (**Figure 7E**). Additionally, we established a positive correlation between these five TFs and macrophage markers Cd86, Cd80, Cd163, Arg1 (**Figure 7F**).

Additionally, datasets CRA001143 and SRP221729 were selected from the integrated dataset for analysis to support our findings. These datasets were prioritized due to their larger sample sizes, thereby mitigating the influence of inter-individual variability on the robustness of the results. **Supplementary Figure 4A** presents immune cell infiltration results for the Ischemia and Sham groups in a publicly available mouse dataset, using a stacked bar chart, revealing macrophages as the predominant responsive cell population to the ischemic insult—results in **Supplementary Figures S4B, C** demonstrate elevated numbers of M0 and M1 macrophages alongside reduced populations of M2 macrophages, various T cell subsets, and monocytes in the Ischemia group (*P* < 0.05, *P* < 0.01, and *P* < 0.001). Correlation analysis of immune cells in the public dataset further revealed interactions among various immune cell types, particularly between macrophages and neutrophils, as well as between distinct T cell subsets, DCs, eosinophils, mast cells, NK cells, B cells, and monocytes (**Supplementary Figure 4D**). These TFs exhibited negative correlations with M2 macrophages, various T cells, and monocytes, and a positive correlation with M0 and M1 macrophages. Furthermore, a positive correlation was established between these five TFs and the macrophage markers Cd86, Cd80, Cd163, Arg1, and NOS2 (**Supplementary Figures S4E, F**).

Collectively, our results demonstrate that macrophages constitute the predominantly altered cell population in the murine cerebral ischemia model. Moreover, the identified orthologous TFs exhibit significant correlations with macrophages.

### Association of key orthologous TFs with immune infiltration in rats

**Figure 8:**
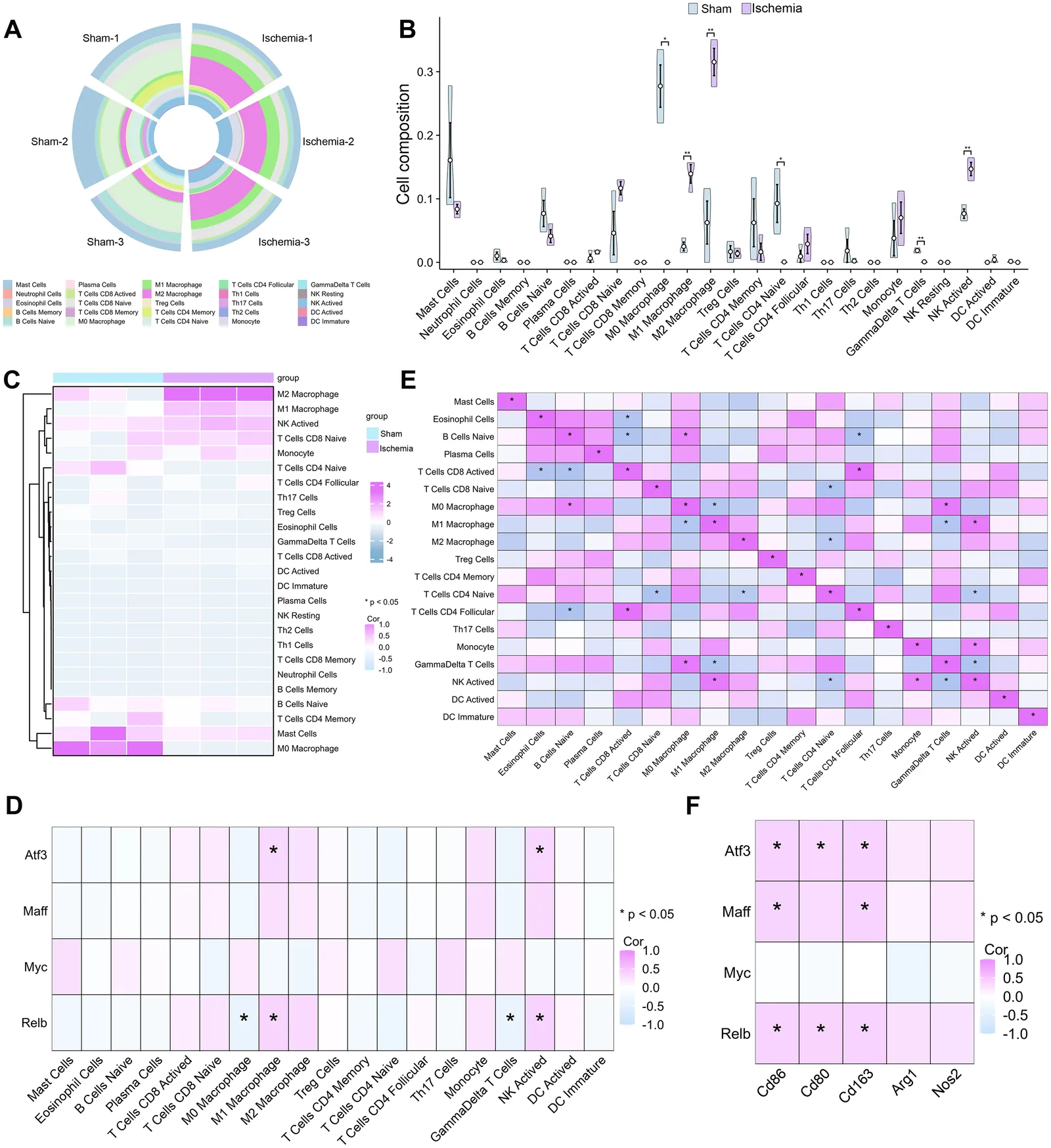
Profiling immune cell infiltration utilizing our self-generated rat dataset. **(A)** Stacked histograms illustrate the extent of immune cell infiltration in different groups. **(B)** Comparison of immune cell infiltration levels between the Sham and Ischemia groups. **(C)** Clustering of immune cell infiltration. **(D)** Correlation between five hub orthologous TFs and immune cells. **(E)** Analysis of correlations among immune cell populations. **(F)** Correlation between five hub TFs and M1/M2 macrophage marker expression. TFs, Transcription factors. Arg1, Arginase 1. NOS2, Nitric oxide synthase 2. *P< 0.05; **P < 0.01.

Immune infiltration analysis was performed in the rat dataset to assess the association between orthologous TFs and immune cells. Stacked bar charts illustrate the immune cell composition in RNA-seq data from neonatal SD rat HIE models (**Figure 8A**). Compared to the Sham group, quantitative analysis of immune cell abundance indicated a significant increase in M1 macrophages, M2 macrophages, and NK cells, alongside a decrease in M0 macrophages and T cells, in the Ischemia group (*P* < 0.05, *P* < 0.01; **Figures 8B, C**). Correlation analysis among immune cells revealed both positive and negative associations between macrophages and various B and T cells, natural killer cells (**Figure 8D**). Within the in-house rat dataset, the TFs *Atf3* and *Relb* were positively correlated with M1 macrophages and NK cells, while showing a negative correlation between Relb and M0 macrophages as well as γδT cells (**Figure 8E**). Further analysis showed that key orthologous TFs were positively correlated with macrophage markers Cd86, Cd80, and Cd163 (**Figure 8F**).

To further validate the reliability of our in-house dataset, immune infiltration analysis was performed using the publicly available dataset SRP148632. Stacked bar charts illustrate immune cell infiltration in the dataset, with macrophages demonstrating notably high abundance (**Supplementary Figure 5A**). Immunocyte abundance analysis revealed reduced Th17 cell content alongside significantly elevated activated DC levels in the Ischemia group compared with the Sham group (*P* < 0.05; **Supplementary Figures 5B, C**). Subsequent correlation analysis of immune cells revealed significant associations among the macrophages of interest, neutrophils, and B cells (**Supplementary Figure 5D**). Additionally, correlation analysis of TFs *Maff*, *Cebpa*, *Myc*, and *Relb* revealed positive correlations with M1 macrophages but negative correlations with T cells (**Supplementary Figure 5E**). Further analysis showed that key orthologous TFs were positively correlated with macrophage markers Cd86 (**Supplementary Figure 5F**).

In conclusion, integrated immune infiltration analyses across rat ischemia models identify macrophages as pivotal mediators of neuroimmune inflammation. Orthologous TFs in these cells may modulate macrophage function or ontogeny, underscoring macrophage-centric research as a critical future direction.

### Orthologous TFs localize primarily to microglia

**Figure 9:**
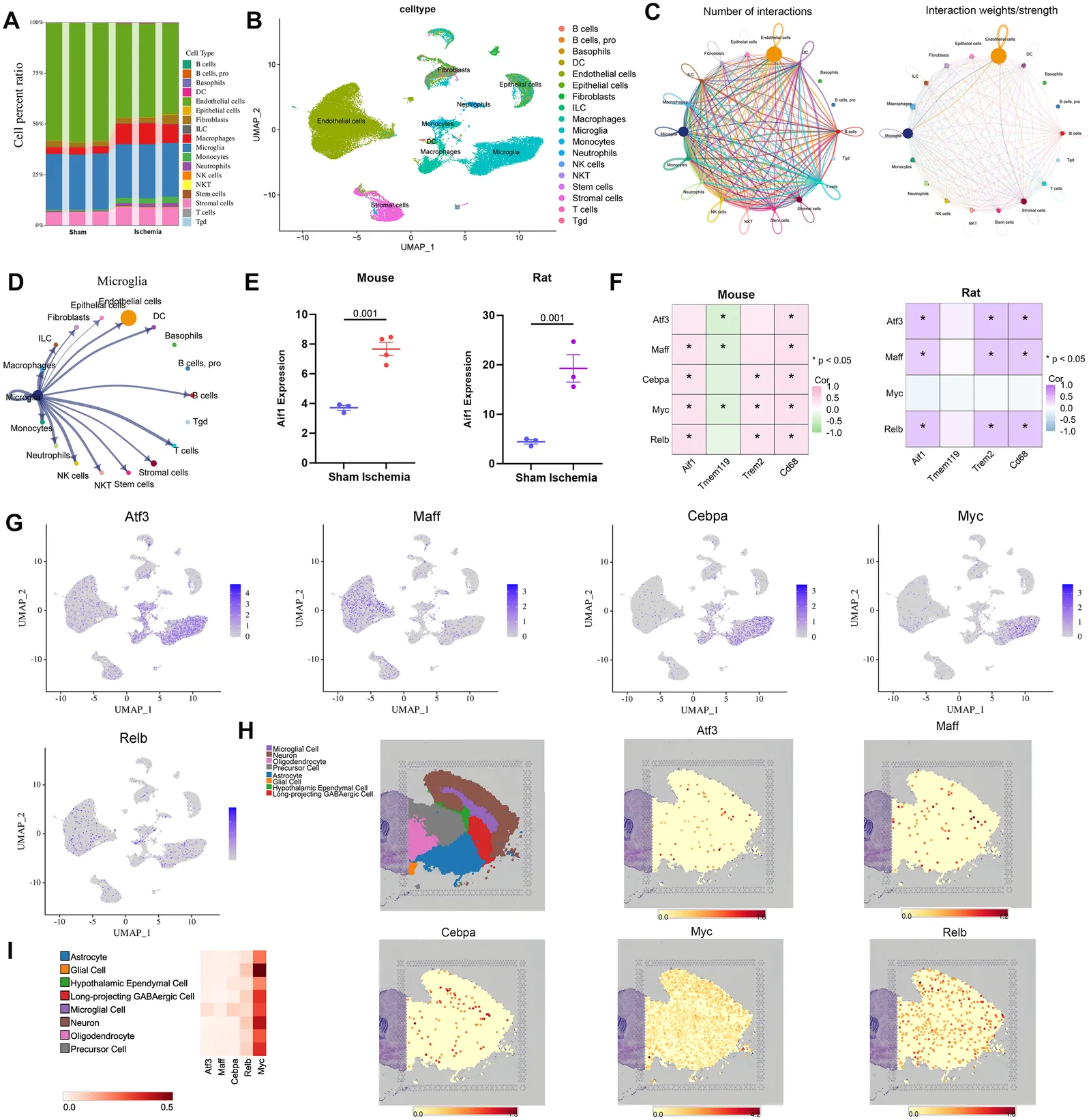
Orthologous TFs may regulate microglial function. **(A)** Distribution of cell clusters in GSE174574. **(B)** UMAP visualization of the single-cell transcriptome atlas. Color indicates cell type. **(C)** Cell-cell communication network for major cell types in GSE174574. Cell types are color-coded; edge thickness denotes interaction strength between sender and receiver pairs. **(D)** Ligand-receptor interaction network for microglia subgroups. Node size represents cell abundance; arrows indicate ligand-receptor interactions, originating from sender cells and pointing to receiver cells. **(E)** The FPKM values of Aif1 in our self-generated mouse and rat dataset. Mouse 95%CI (2.543-5.360); Rat 95%CI (7.035-22.64). **(F)** Correlation analysis of key TFs and microglia marker Aif1/Iba1, Tmem119, Trem2, and Cd68 in the self-test dataset. **(G)** UMAP plots showing expression distribution of *Atf3*, *Maff*, *Cebpa*, *Relb*, and *Myc* signature in single cell clusters. **(H, I)** 10X Visium spatial scatter pie plot representing the proportions of the cells from the reference atlas within capture locations in brain tissue in mice, seven days after birth. Predicted proportion within each capture location for the hub TFs. UMAP, Uniform manifold approximation and projection. FPKM, Fragments per kilobase of exon model per million mapped fragments. Aif1/Iba1, Allograft inflammatory factor 1/Ionized calcium-binding adapter molecule 1; Tmem119, transmembrane protein 119; Trem2, triggering receptor expressed on myeloid cells-2. Cd68, Cluster of Differentiation 68; TFs, Transcription factors. *P < 0.05.

To determine the distribution of these five key TFs across different cell types in stroke samples, we analyzed the single-cell RNA sequencing dataset GSE174574. The results of cell clusters and cell proportions are shown in **Figures 9A, B**. CellChat analyzed intercellular communication among different immune cell types in the ischemic stroke environment. As a unique type of macrophage in the brain, microglia interact extensively with various immune cells (**Figures 9C, D**). Meanwhile, in our RNA sequencing datasets from both adult mouse MCAO and neonatal rat HIE models, microglial markers Aif1 (Iba1) and Trem2 showed higher FPKM values in the Ischemia group than in the Sham group (*P* < 0.05, *P* < 0.01, *P* < 0.001; **Figure 9E**; **Supplementary Figures 6A, B**). Correlation analysis revealed a close relationship between the key conserved TFs and microglia (**Figure 9F**; **Supplementary Figures 6C, D**), suggesting their potential involvement in regulating microglial function in response to ischemic insults.

Additionally, we used density plots to show the distribution of expression levels for the *Atf3*, *Maff*, *Cebpa*, *Myc*, and *Relb* TFs in single cells. The results in **Figure 9G** indicate that *Atf3*, *Cebpa*, *Myc*, and *Relb* are primarily expressed in microglia. Furthermore, spatial transcriptomic data from postnatal day 7 mouse brain tissue provided supportive baseline expression patterns that are broadly consistent with the microglial localization observed by immunofluorescence in our ischemic models (**Figures 9H, I**). It should be noted that the spatial transcriptomic dataset used for this analysis originates from normal postnatal day 7 mouse brain, not from ischemic tissue. Therefore, it can only confirm baseline anatomical distribution of the identified TFs; its utility for validating ischemia-induced spatial expression changes is limited. Taken together, our findings provide supporting evidence for the reliability of immune infiltration analysis and reveal that orthologous TFs may respond to cerebral ischemic challenge by modulating microglial function, highlighting their potential as biomarkers.

### Cebpa and Relb serve as signature TFs for ischemic stroke

**Figure 10:**
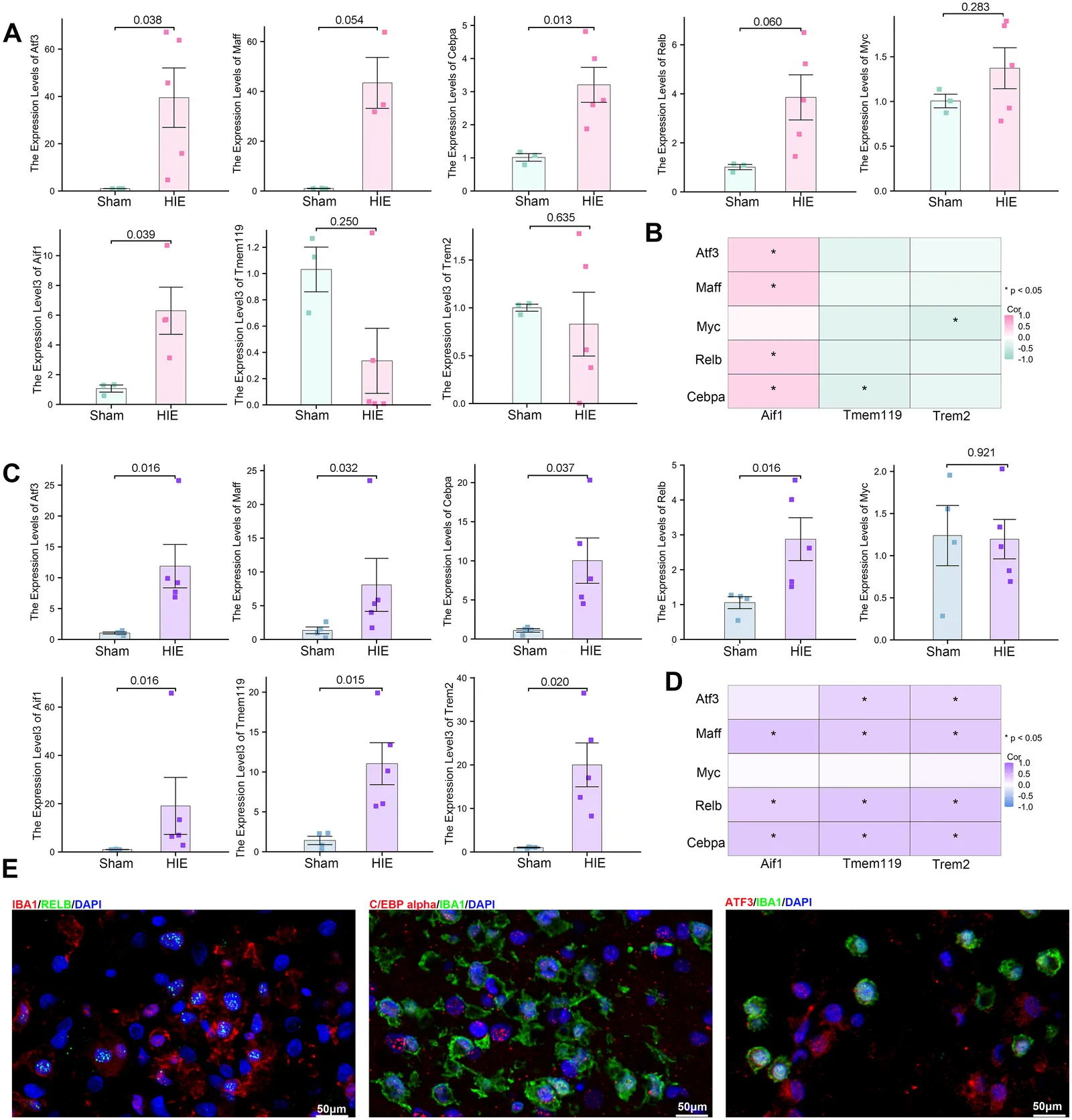
*In vivo* validation of the association between orthologous TFs and microglia. **(A)** qRT-PCR analysis of key orthologous TFs and microglia markers Aif1/Iba1, Tmem119, and Trem2 in the HIE group at day 1 post-reperfusion. The 95% CIs for the relative expression levels were as follows: *Atf3*: 3.5338-73.372; *Maff*: -1.5753- 86.372; *Cebp*a: 0.74032-3.6433; *Relb*: -0.1645-5.848; *Myc*: -0.39406-1.1258;*Aif1*: 0.38728- 10.079; *Tmem119*: -1.2582-0.59324; *Trem2*: -1.0939-0.75019. qRT-PCR was performed with 3–5 biological replicates and 2 technical replicates per sample; Ct values were averaged. **(B)** Correlation analysis between hub TFs and Aif1/Iba1, Tmem119, Trem2 in the HIE group at day 1 post-reperfusion. **(C)** qRT-PCR validation of key orthologous TFs and microglia markers Aif1/Iba1, Tmem119, and Trem2 mRNA expression in the 3-day post-injury model. The 95% CIs for the relative expression levels were as follows: *Atf3*: 5.7324-24.731; *Maff*: 0.18104-22.574; *Cebp*a: 0.89148- 16.969; *Relb*: 0.28018-3.4671; *Myc*: -1.014-0.92978;*Aif1*: 1.8192-64.833; *Tmem119*: 2.4777-16.76; *Trem2*: 5.0149-32.981. **(D)** Heatmap analysis of the correlation between hub TFs and microglia markers in the 3-day post-injury model. **(E)** *Cebpa* and *Relb* predominantly co-localize with microglia, whereas *Atf3* shows minimal co-localization. Bar=50 µm. TFs, Transcription factors. Aif1/Iba1, Allograft inflammatory factor 1/Ionized calcium-binding adapter molecule 1; Tmem119, Transmembrane protein 119; Trem2, triggering receptor expressed on myeloid cells-2. HIE, Hypoxic-ischemic encephalopathy; qRT-PCR, quantitative real-time polymerase chain reaction. *P< 0.05.

To validate the bioinformatics findings, we established a rat model of neonatal HIE and collected brain tissue samples at 1 day and 3 days post-reperfusion. Elevated expression of key TFs and the microglial/macrophage marker Iba1 was observed in the HIE group at day 1 post-reperfusion. Specifically, the expression levels of *Atf3*, *Cebpa*, and *Relb* were significantly increased (*P* < 0.05), as were the levels of Aif1 (Iba1) (*P* < 0.05). Importantly, significant positive correlations were detected only between the expression levels of *Atf3*, *Maff*, *Cebpa*, *Relb*, and *Aif1* (**Figures 10A, B**). In the 3-day post-injury model, expression levels of the core TF *Atf3*, *Maff*, *Cebpa*, *Relb*, as well as Aif1, Tmem119, and Trem2, were significantly elevated in the HIE group compared to the Sham group (*P* < 0.05). Furthermore, these factors demonstrated significant positive correlations (**Figures 10C, D**). To further validate these findings, we performed co-localization experiments analyzing the key TFs *Atf3*, *Cebpa*, and *Relb*, with Iba1^+^ microglial/macrophage cells in rat models at 3 days post-reperfusion. As demonstrated in **Figure 10E**, the TFs *Cebpa* and *Relb* exhibited robust co-localization with Iba1^+^ microglial/macrophage cells. In contrast, only a minor fraction of the *Atf3* signal overlapped with this cell type. These experimental findings validate the reliability of our prior bioinformatic predictions and indicate that the two orthologous key TFs, *Cebpa* and *Relb*, represent promising biomarker candidates for ischemic stroke in rodent models.

## Discussion

In this study, robust DEGs in rodent models were identified and found to be enriched in immune cell differentiation and immune response pathways. From these DEGs, 51 orthologous TFs enriched in leukocyte differentiation were further screened. Machine learning identified a biomarker panel—*Atf3*, *Maff*, *Cebpa*, *Relb*, and *Myc*. Immune infiltration analysis, qRT-PCR, and immunofluorescence staining revealed that *Cebpa* and *Relb* significantly co-localize with macrophages (microglia), suggesting their role in post-ischemic inflammation or repair. Notably, *Cebpa* and *Relb* were identified as key microglial TFs linked to neuroimmune dysregulation. Collectively, these findings highlight the potential of these TFs as biomarkers and therapeutic targets for assessing ischemic injury severity.

Unlike previous studies relying on limited datasets (23, 24), this study integrated 10 independent datasets covering multiple species, injury models, and post-ischemic time points. This systematic approach enabled reliable identification of robust genes under heterogeneous conditions, overcoming limitations of single-model or single-timepoint analyses—such as species-specific bias and transient responses. Multidimensional integration and cross-validation enhanced statistical power and biological credibility, allowing precise distinction between background noise and core genes consistently dysregulated across injury contexts. Subsequent enrichment analysis of stably dysregulated genes revealed that, in both rats and mice, upregulated genes were significantly enriched in immune response pathways, whereas downregulated genes primarily converged on neuronal synaptic signaling functions, consistent with previous reports (23, 24). This evolutionarily conserved functional pattern underscores the importance of prioritizing immune response and synaptic signaling pathways in future research.

Previous studies have shown that RelB and C/EBPα regulate Peyer’s patch mononuclear phagocyte development (25) and promote macrophage maturation and antitumor responses (26), consistent with our findings. To validate these orthologous TFs, we assessed their expression in brain ischemia models across different ages. In the HIE model, *Atf3*, *Cebpa*, and *Relb* were significantly upregulated and positively correlated with microglial markers, reinforcing their therapeutic potential. *Atf3*, an early-response gene in the CREB family (27), is a well-established marker of cellular stress and injury (28), with low basal expression and rapid induction under pathological conditions (29). In the peripheral nervous system, *Atf3* is strongly upregulated in sensory and motor neurons after injury (27, 30) and promotes axonal regeneration (31, 32). Accumulating evidence supports *Atf3* as a neuron-specific biomarker in CNS and PNS injuries. A 2024 study reported increased neuronal *Atf3* co-localization after spinal cord injury or ischemic stroke (33) and single-cell RNA-seq data confirm its upregulation in sensory and motor neurons following peripheral nerve injury (34, 35). Consistent with these reports, our qRT-PCR analysis showed increased *Atf3* expression in the ischemic cortex. However, immunofluorescence staining revealed minimal co-localization with microglial markers, diverging from our computational prediction but aligning with the view that *Atf3* is primarily neuronal. Moreover, external validation showed an AUC below 0.7 for *Atf3*, indicating low diagnostic specificity and further supporting that *Atf3* may not be a principal regulatory TF in rodent microglia.

The findings of this study have multiple potential implications for clinical and public health services. First, the orthologous transcription factors identified in rodent cerebral ischemia models demonstrated stable diagnostic efficacy and may serve as auxiliary biomarkers for human ischemic stroke, providing a theoretical basis for translating rodent findings to clinical applications. Second, *Cebpa* and *Relb* were validated as key microglial transcription factors involved in neuroimmune regulation; targeting these factors could modulate microglial function to suppress excessive inflammation and promote neural repair, offering novel therapeutic strategies for ischemic stroke. Previous studies have reported that knocking down *Cebpb* inhibits neuronal apoptosis and improves neurological recovery in mice. Third, from a public health perspective, given the high morbidity, disability, and mortality of ischemic stroke, the identified biomarkers and therapeutic targets may reduce stroke-related disability and mortality through diagnosis and targeted intervention, thereby alleviating societal healthcare burdens. This research paradigm may also inform translational studies of other neurological disorders. However, despite the promising diagnostic performance of *Cebpa* and *Relb* in rodent tissue-based models, their intracellular localization and the presence of the blood–brain barrier pose significant challenges for direct peripheral detection. Nonetheless, transcription factors can be released into circulation via extracellular vesicles (36). Future studies should explore whether *Cebpa* and *Relb* are detectable in plasma-derived exosomes or cerebrospinal fluid following cerebral ischemia, enabling non-invasive prognostic stratification or monitoring of neuroimmune activation. These TFs may also serve as therapeutic targets, given that small-molecule modulators of *Cebpa*/*Relb* pathways are under development for inflammatory diseases. We emphasize that these translational pathways are speculative and require extensive validation in human samples before clinical application.

This study has several limitations. (1) Although quality control and standardized processing were performed on the datasets, the inability to fully trace or verify the original data collection processes, experimental operation details, and sample quality control status may potentially affect the reliability of the analytical results. (2) While preliminary PCA revealed clear separation between groups with no apparent batch effects across datasets, it must be acknowledged that undetected technical biases may still exist. (3) The dynamic expression patterns and temporal regulatory characteristics of transcription factors at different post-injury time points were not systematically examined. (4) We acknowledge that CIBERSORT-based deconvolution cannot distinguish resident microglia from infiltrating macrophages in brain tissue. Consequently, our immune infiltration analysis could not determine whether the key orthologous transcription factors reflect M1-driven pro-inflammatory processes, M2-mediated tissue repair, or their dynamic interplay across different disease stages. These findings should therefore be interpreted as exploratory and warrant future validation using brain-specific immune reference signatures or single-cell technologies. (5) Given the inherent risks of translating findings from rodent models to clinical applications, validation in human samples is required to confirm the potential clinical value of our conclusions. (6) Correlation analysis cannot establish causality, and the biological interpretation of enrichment analysis is inherently dependent on the completeness and accuracy of the databases used. To address these limitations and elucidate functional significance, future studies should: (1) conduct large-scale multicenter clinical cohort studies to control data heterogeneity at the source and minimize technical biases; (2) perform longitudinal studies with continuous time-point sampling to reconstruct the dynamic regulatory networks of transcription factors during ischemic injury progression; (3) Future studies should employ high-resolution techniques (37, 38), such as spatial transcriptomics or single-cell sequencing, in combination with co-staining of TMEM119/P2RY12 and CCR2/Ly6C, to resolve microglial expression profiles across distinct polarization states and to further verify the precise localization and functional roles of both resident microglia and infiltrating macrophages in the injured brain; and (4) utilize gain- and loss-of-function experiments to validate the causal roles of key transcription factors and elucidate their exact mechanisms in neuroimmune regulation, providing deeper insights into their functions in cerebral ischemic injury and repair.

## Conclusions

This study provides the first comprehensive profile of orthologous TFs associated with cerebral ischemia in mice and rats, focusing on immune infiltration and injury response. A screening process identified several previously understudied orthologous TFs (including *Atf3*, *Maff*, *Cebpa*, *Relb*, and *Myc*) as being linked to ischemic brain injury and immune infiltration. Nevertheless, only *Cebpa* and *Relb* demonstrated significant associations and spatial co-localization with macrophages/microglia, suggesting their involvement in neuroimmune dysregulation. These findings provide a theoretical foundation for identifying *Cebpa* and *Relb* as potential ischemia-related microglial TFs in rodent models, providing a theoretical foundation for future mechanistic studies of ischemic brain injury.

## Data Availability

The datasets presented in this study can be found in online repositories. The names of the repository/repositories and accession number(s) can be found in the article/**Supplementary Material**.

## Supplementary material

Supplementary Figure 1 Merging datasets and removing batch effects. **(A)** Box plot showing the sample distribution of each data set after batch correction. **(B)** UMAP analysis showing the sample distribution of each dataset after batch correction. **(C)** PCA results of the datasets after batch correction. UMAP, Uniform Manifold Approximation and Projection; PCA, Principal Component Analysis.

Supplementary Figure 2 Heatmap of stably up- and down-regulated DEGs in rodents. **(A, B)** Heatmap of robustly up- and down-regulated DEGs in mice. **(C, D)** Heatmap of robustly up- and down-regulated DEGs in rat models. DEGs, differentially expressed genes.

Supplementary Figure 3 Harmonization of the data. **(A)** Box plots showing the sample distribution of each dataset after batch effect removal in the in-house dataset. **(B)** PCA results after batch correction in the in-house dataset. PCA, Principal Component Analysis.

Supplementary Figure 4 Analysis of immune infiltration for key orthologous TFs in mouse public datasets. **(A)** Stacked bar chart showing the distribution of immune cells in the Sham and Ischemia groups. **(B)** Comparison of immune cell infiltration levels in the Sham and Ischemia groups from the public dataset. **(C)** Heatmap displays immune cell expressions in different groups across the two datasets. **(D)** Heatmap illustrating correlations among immune cells in the public dataset. **(E)** Analysis of the correlation between core TFs and various immune cell types. **(F)** Correlation analysis of expression levels of 5 key TFs with M1 and M2 macrophage markers. TFs, Transcription factors. **P* < 0.05, ****P* < 0.001.

Supplementary Figure 5 Immune infiltration analysis of core orthologous TFs in public rat datasets. **(A)** Stacked bar chart showing the distribution of immune cells in the Sham and Ischemia groups. **(B)** Comparison of immune cell infiltration between Sham and Ischemia groups using the SRP148632 dataset. **(C)** Heatmap showing correlations among immune cells. **(D)** Heatmap depicting correlations between immune cells within the SRP148632 dataset. **(E)** Correlation of core TFs with immune cell types. **(F)** Analysis of correlations between the expression of 5 hub TFs and M1/M2 macrophage markers. TFs, Transcription factors. **P* < 0.05.

Supplementary Figure 6 Potential relationship between core TFs and microglia. **(A, B)** FPKM values of microglial markers Tmem119 and Trem2 in our mouse MCAO and rat HIE datasets. The 95% CIs for the between-group differences are as follows: in the mouse MCAO model, Tmem119: –3.904 to 0.8903, Trem2: 0.5857 to 5.669; in the rat HIE model, Tmem119: –0.8859 to 1.474, Trem2: –0.5955 to 21.83. **(C, D)** Heatmap showing correlations between key TFs (*Atf3*, Maff, Cebpa, Relb, Myc) and microglial markers (Tmem119, Trem2) in public datasets. TFs, Transcription factors; FPKM, Fragments per kilobase of exon model per million mapped fragments; MCAO, Middle Cerebral Artery Occlusion; HIE, Hypoxic-ischemic encephalopathy. **P* < 0.05.
