# Supplementary material for: Integrated machine learning and transcriptomics reveal immune infiltration-related orthologous transcription genes in cerebral ischemic injury
Source: Front Immunol. 2026 Aug 11;17:1700109. doi: 10.3389/fimmu.2026.1700109 (PMC13503618; doi:10.3389/fimmu.2026.1700109)

cor_matHeatmap_CRA001143

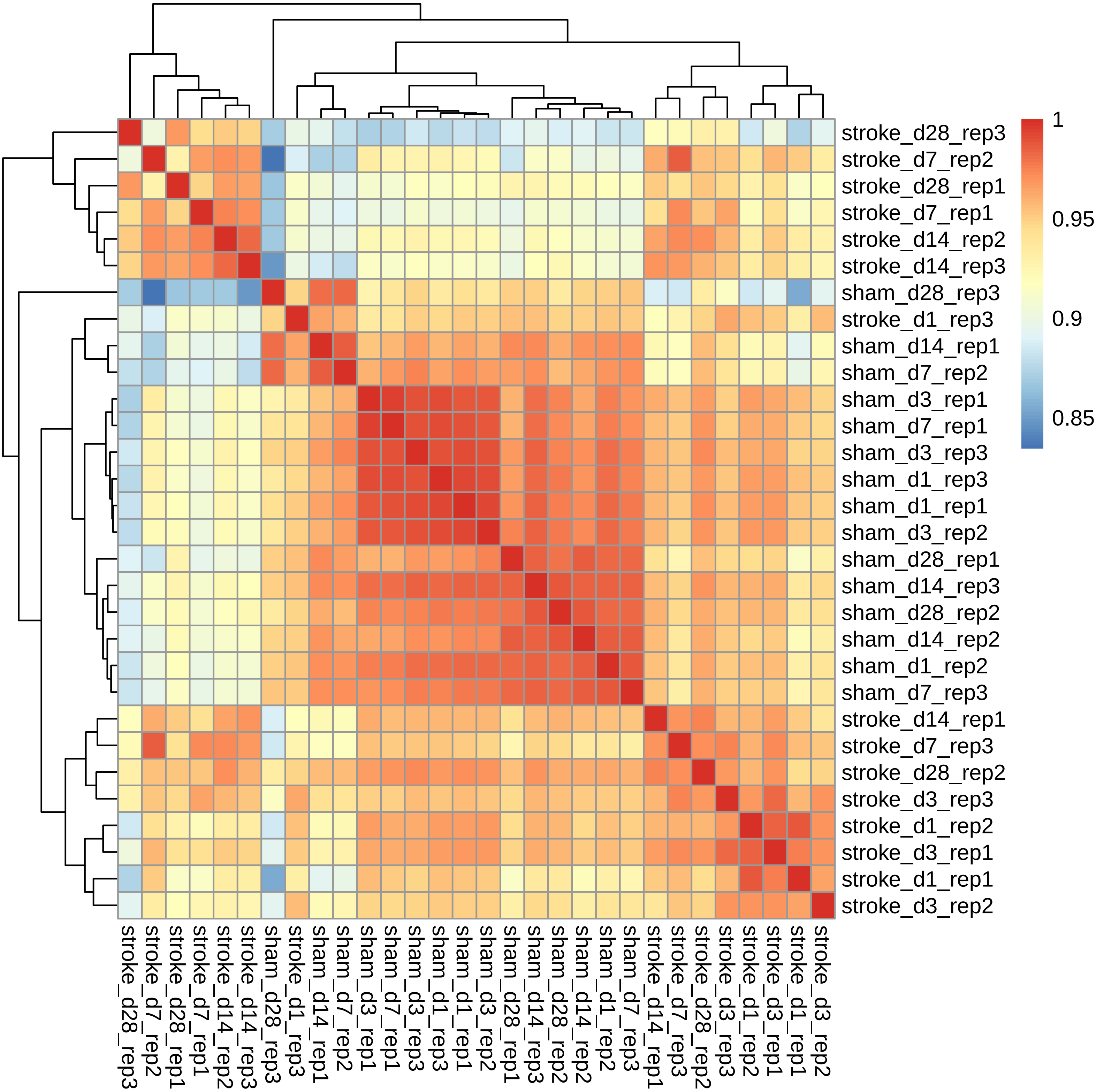

sample_pca_CRA001143

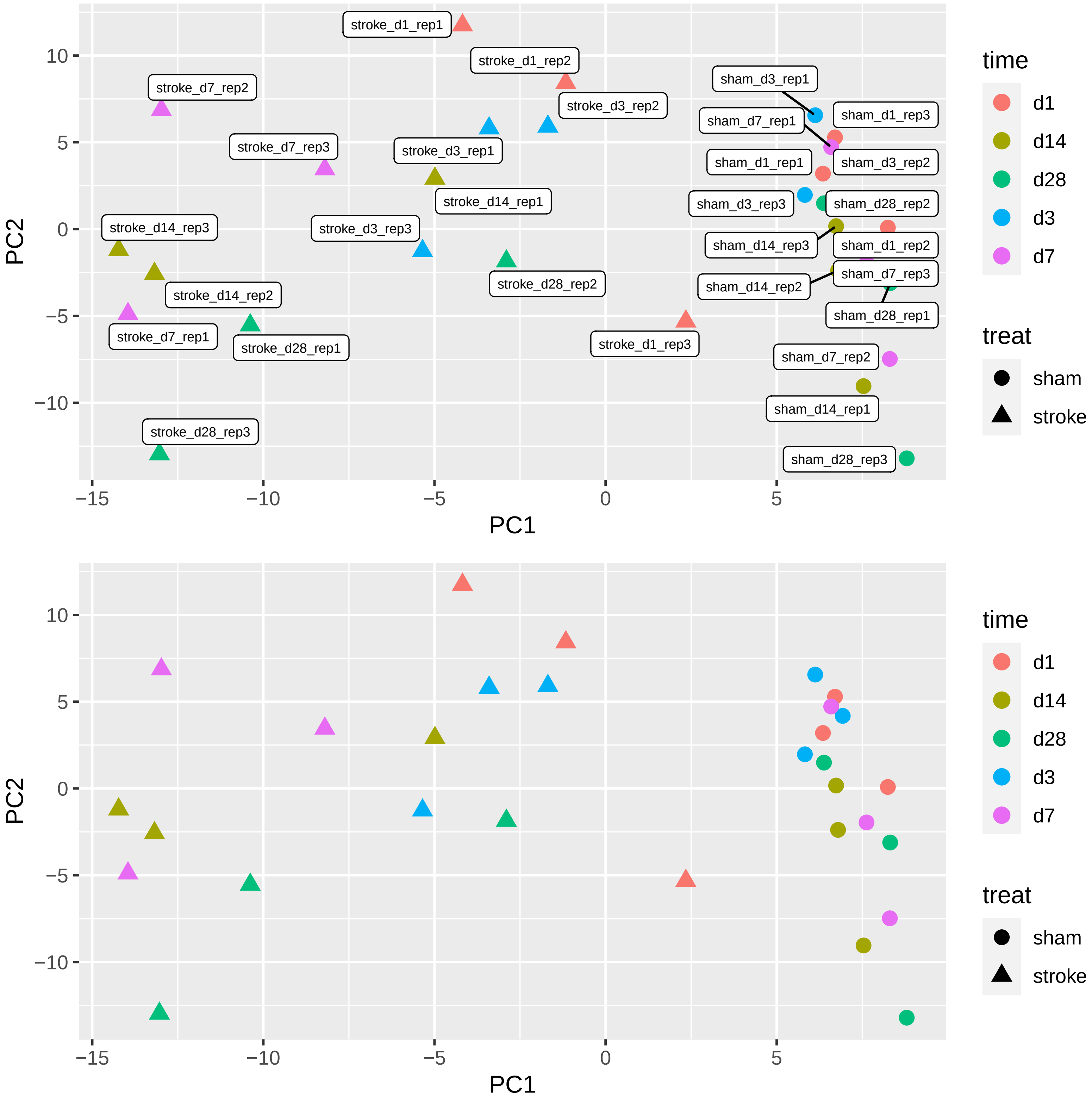

cor_matHeatmap_SRP136487

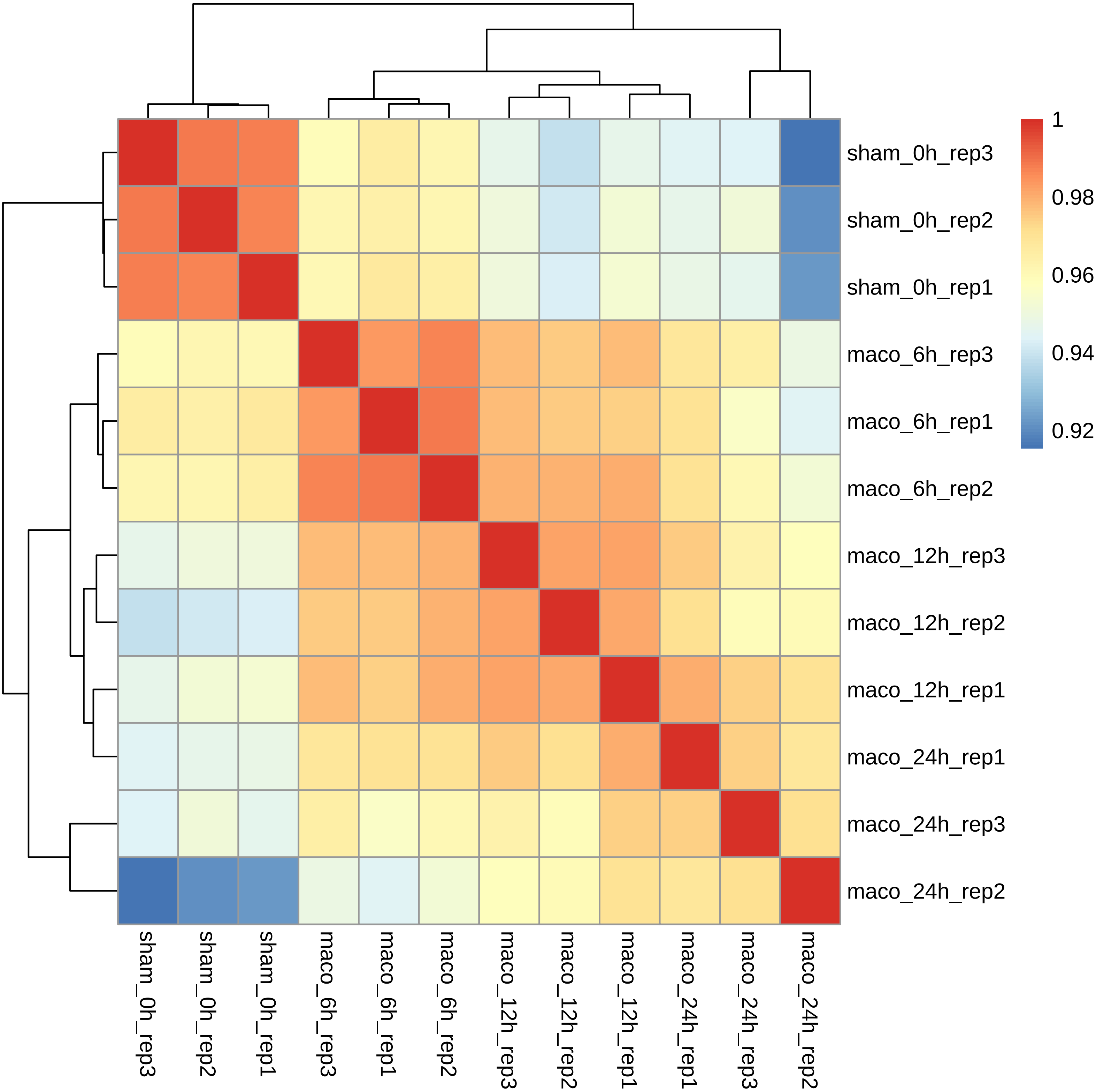

sample_pca_SRP136487

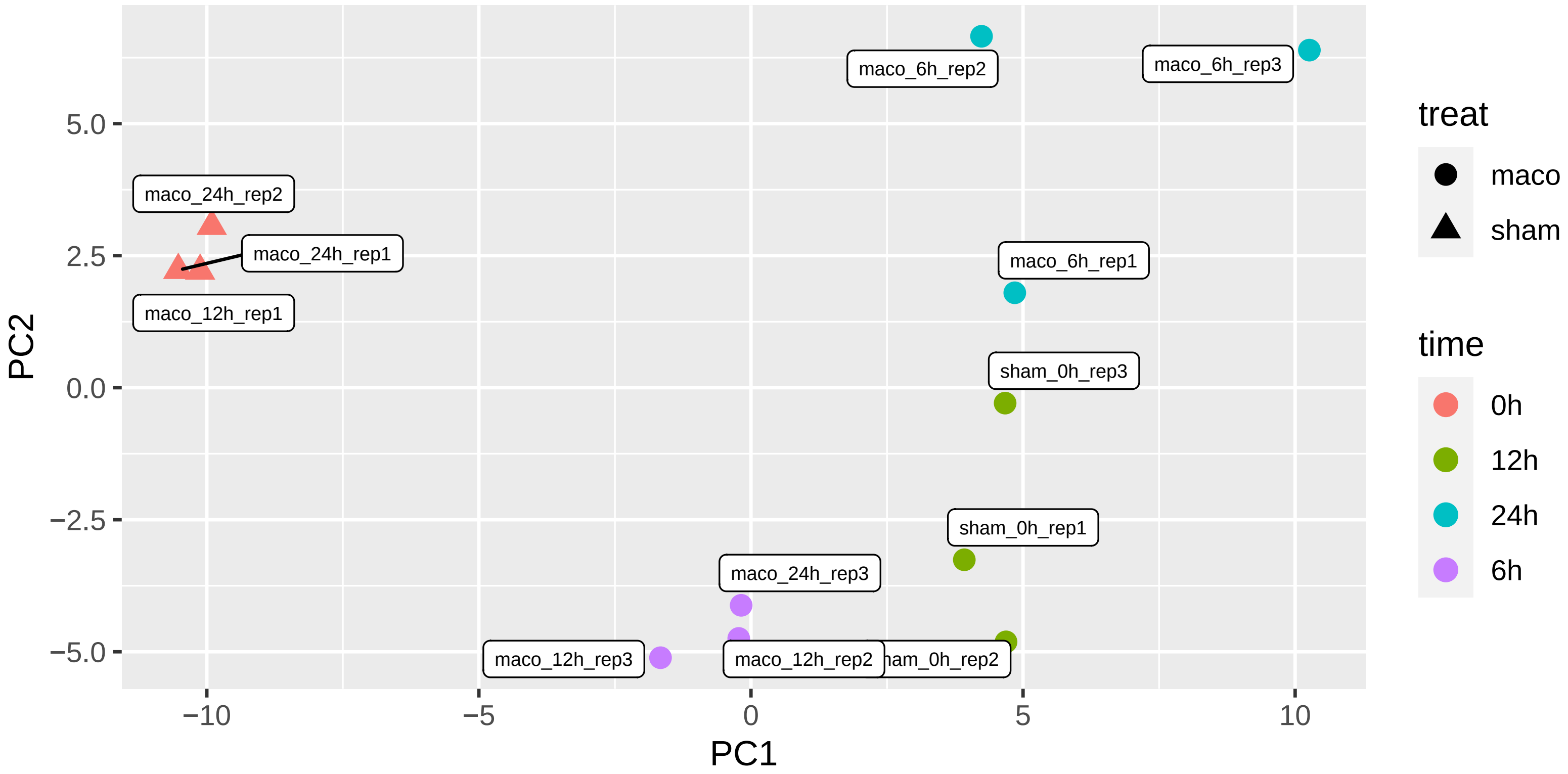

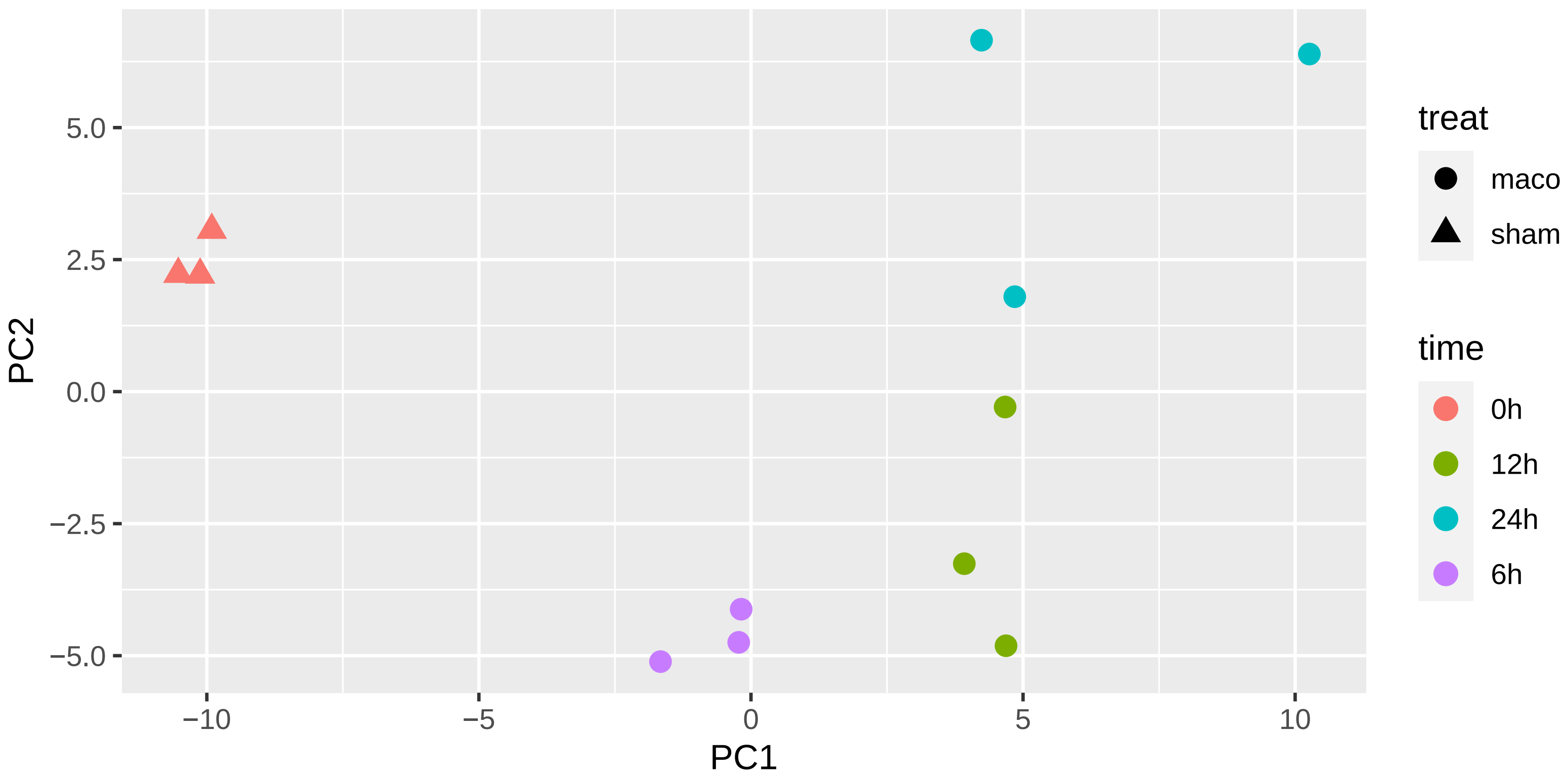

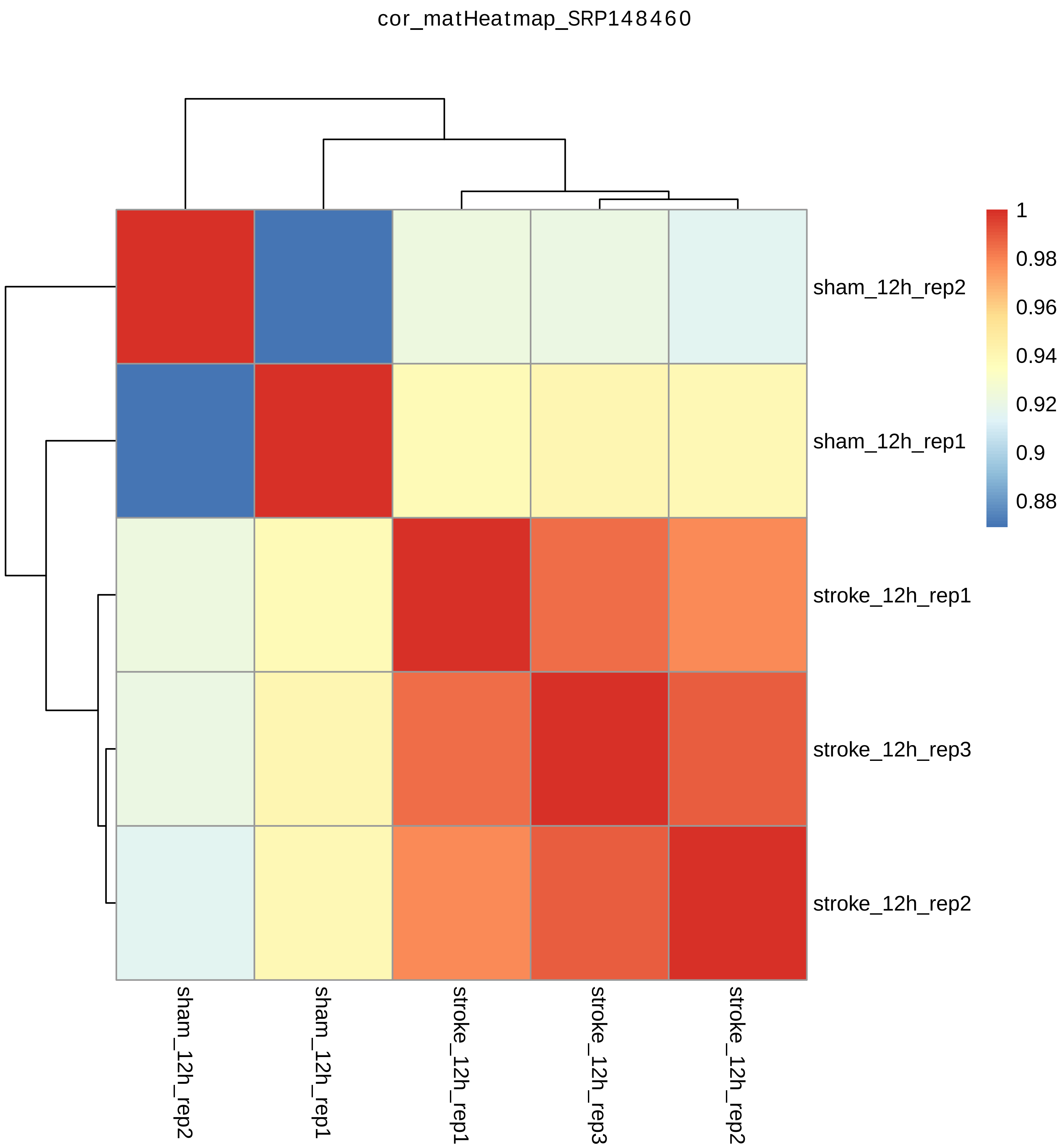
cor_matHeatmap_SRP148460
sham_12h_rep2
sham_12h_rep1
stroke_12h_rep1
stroke_12h_rep3
stroke_12h_rep2
1
0.98
0.96
0.94
0.92
0.9
0.88

sample_pca_SRP148460

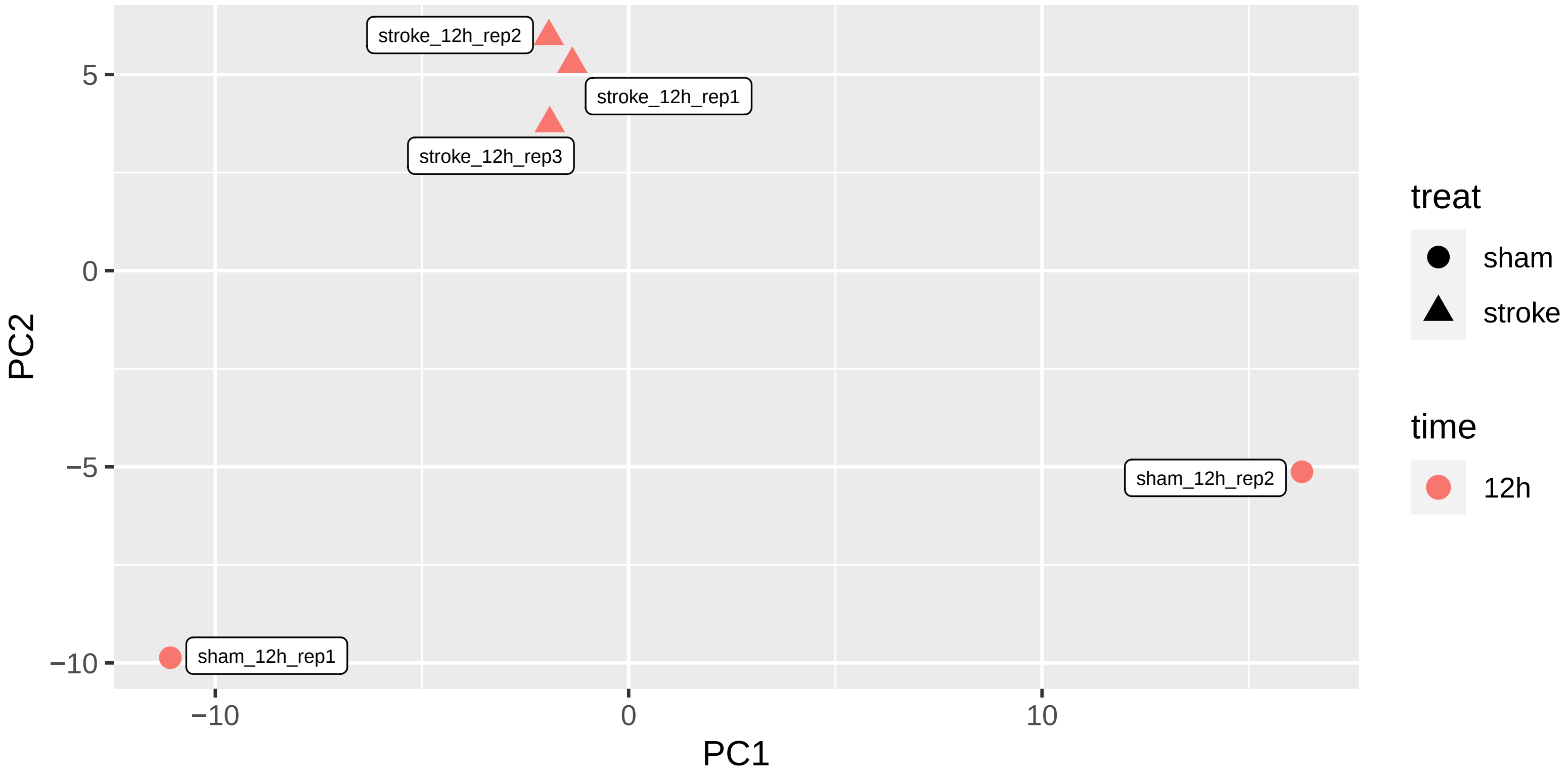

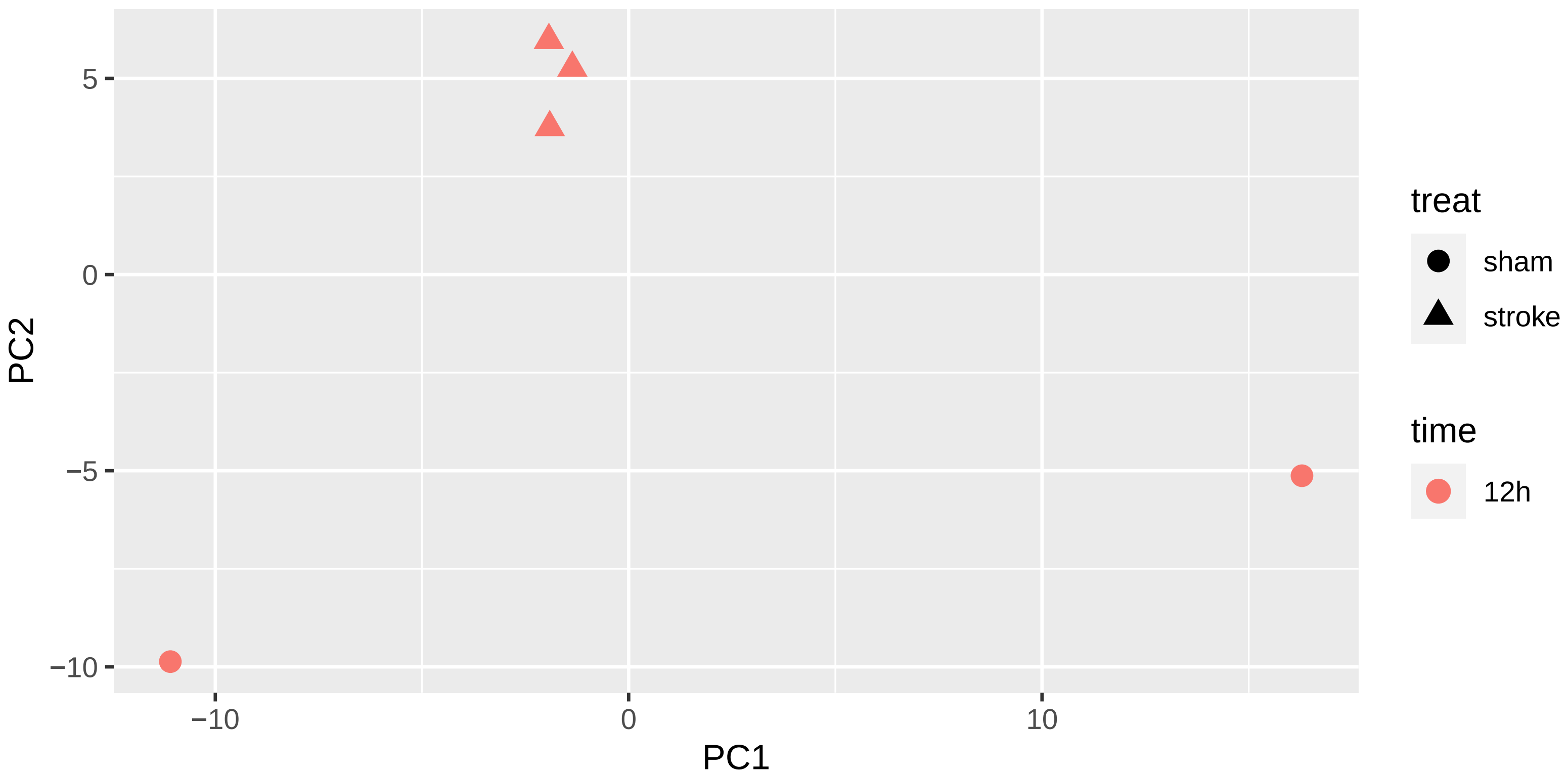

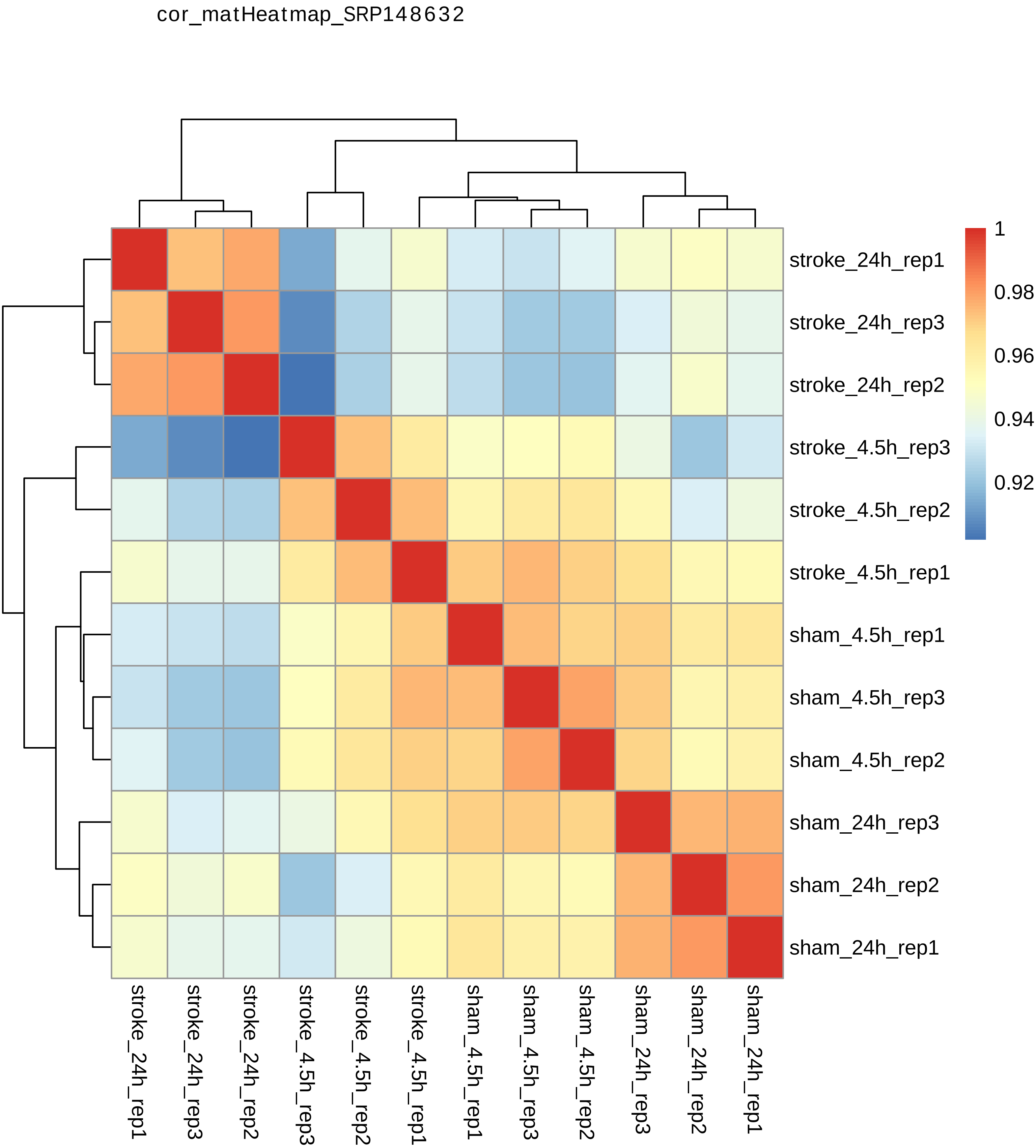
cor_matHeatmap_SRP148632
stroke_24h_rep1
stroke_24h_rep3
stroke_24h_rep2
stroke_4.5h_rep3
stroke_4.5h_rep2
stroke_4.5h_rep1
sham_4.5h_rep1
sham_4.5h_rep3
sham_4.5h_rep2
sham_24h_rep3
sham_24h_rep2
sham_24h_rep1
1
0.98
0.96
0.94
0.92

sample_pca_SRP148632

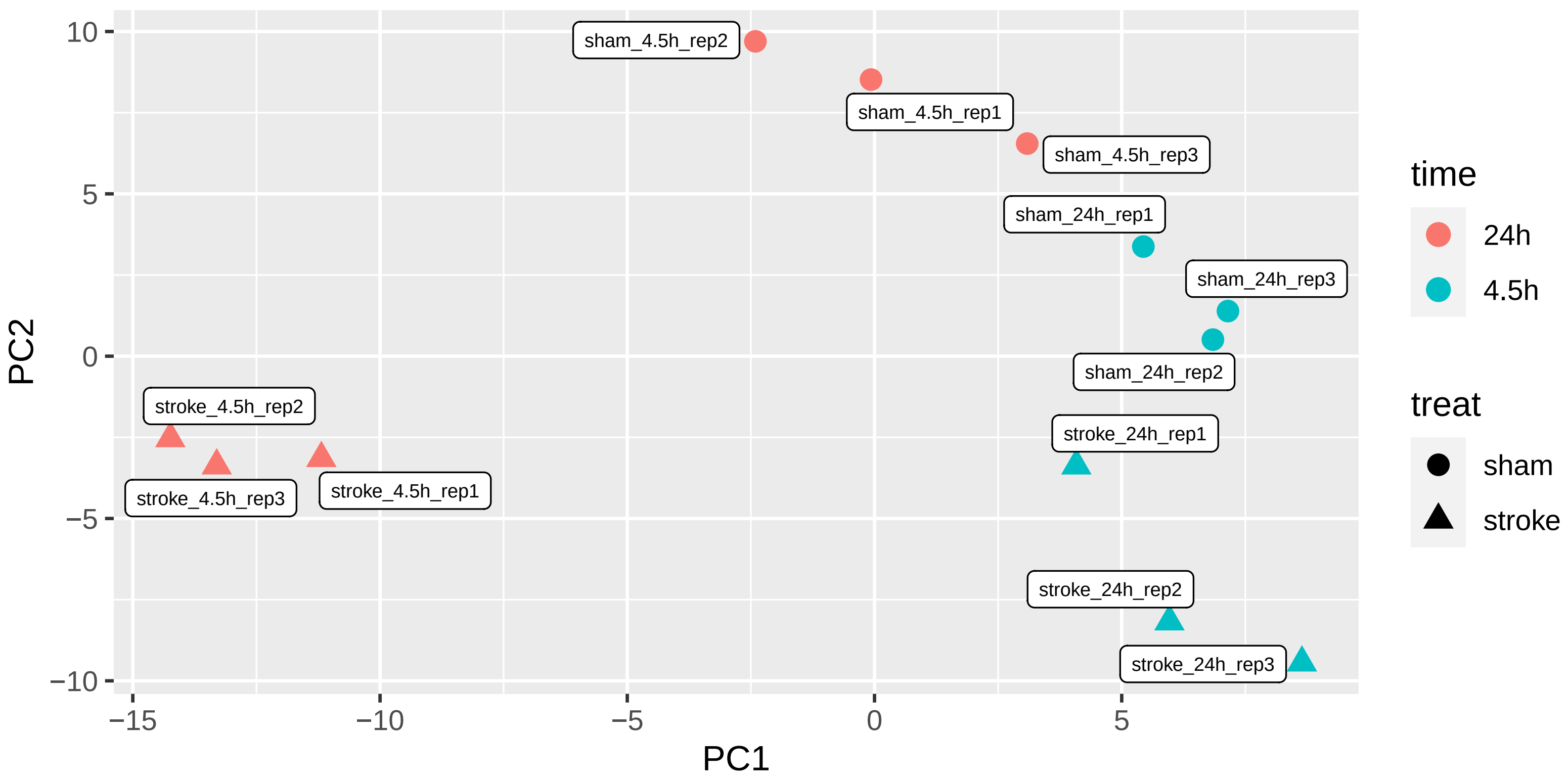

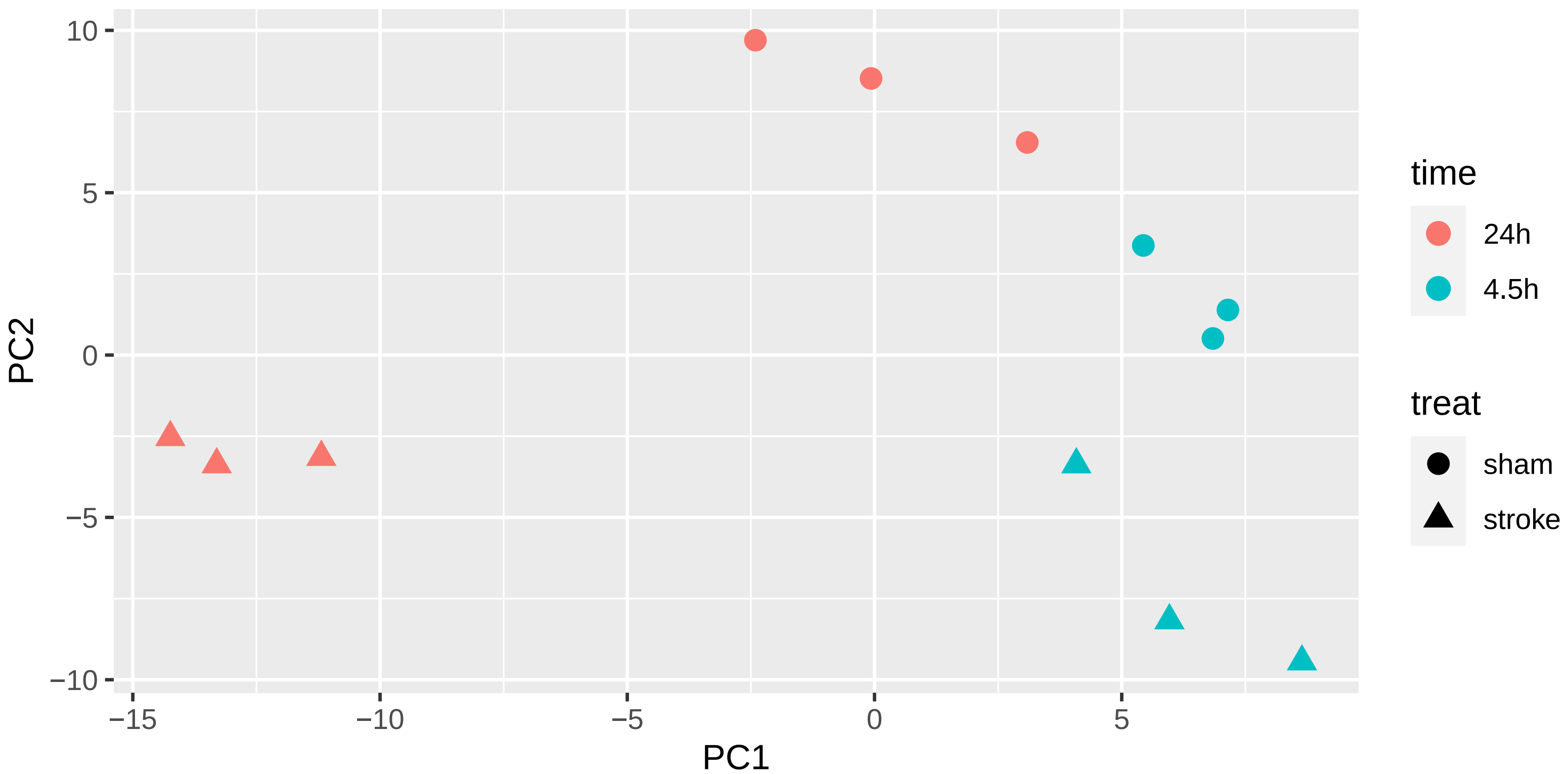

cor_matHeatmap_SRP152966

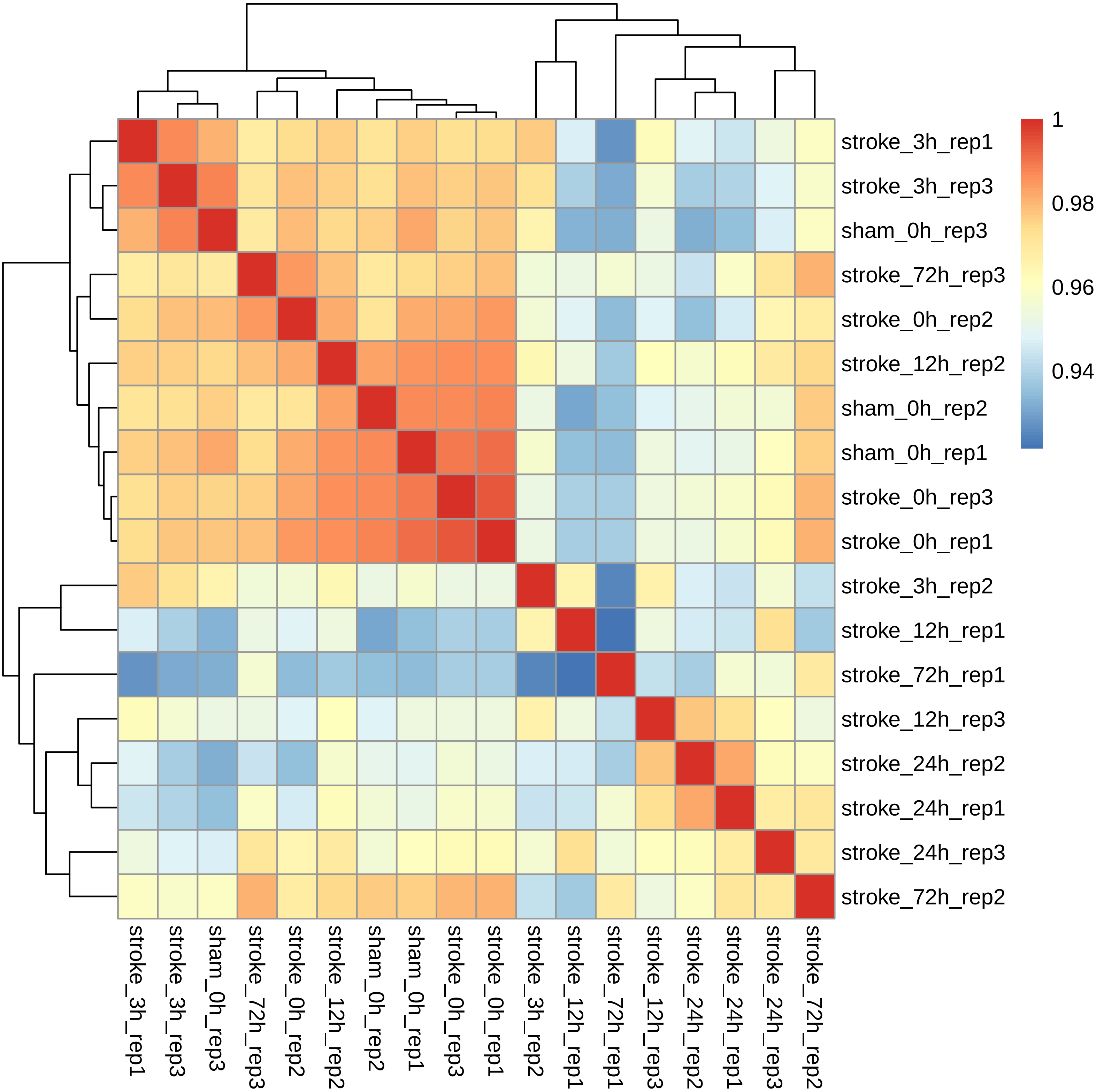

sample_pca_SRP152966

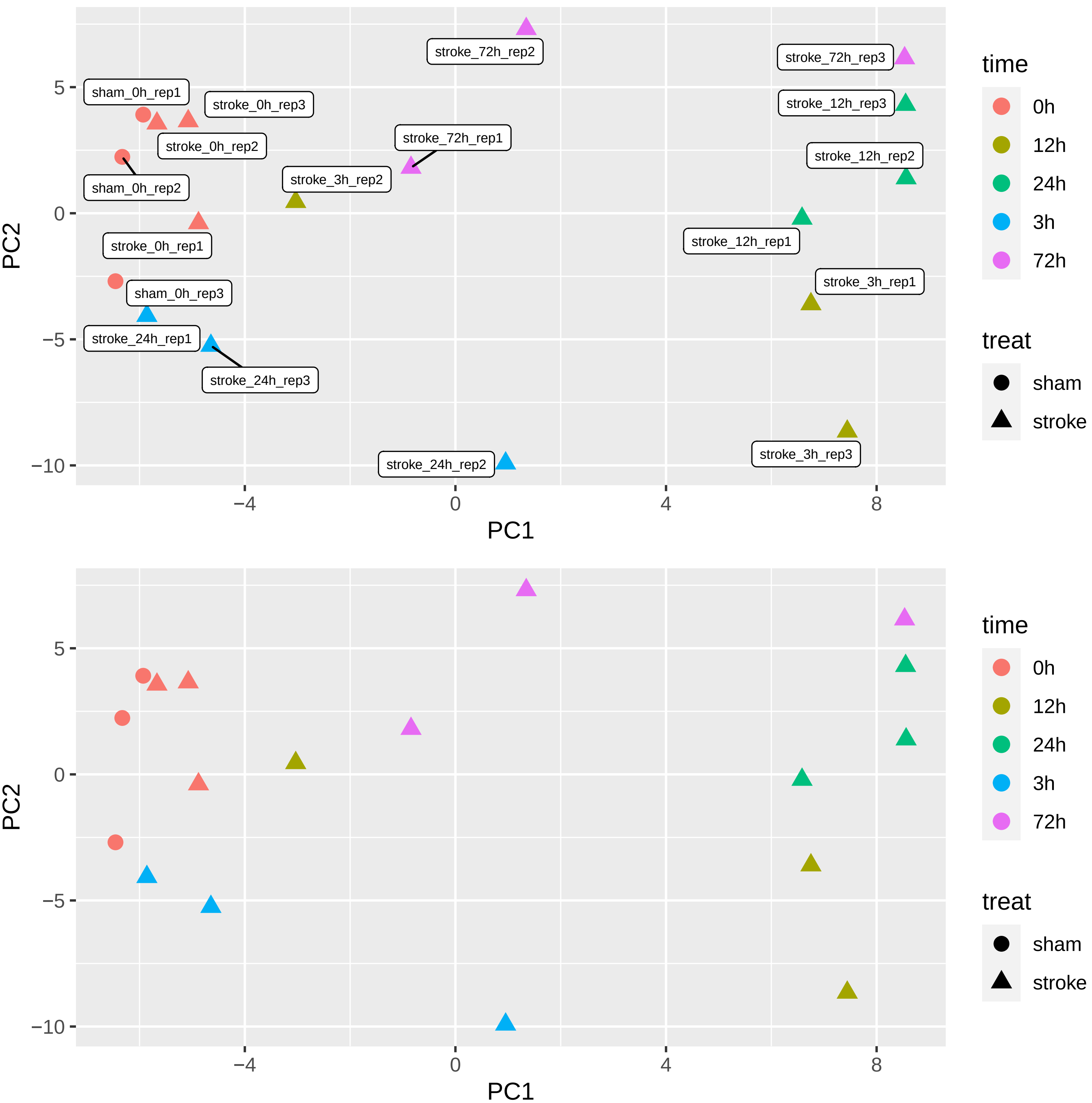

cor_matHeatmap_SRP221729

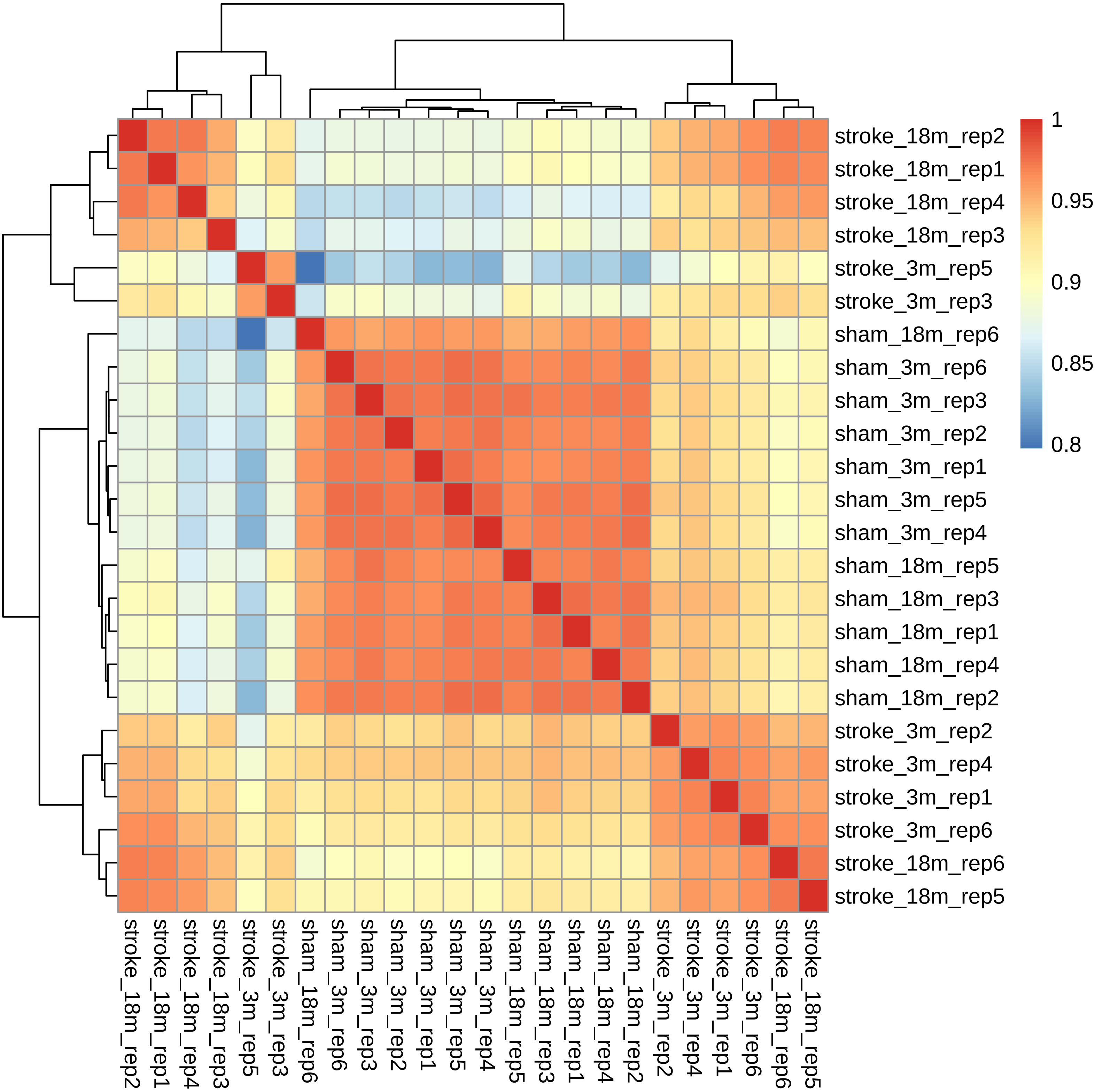

sample_pca_SRP221729

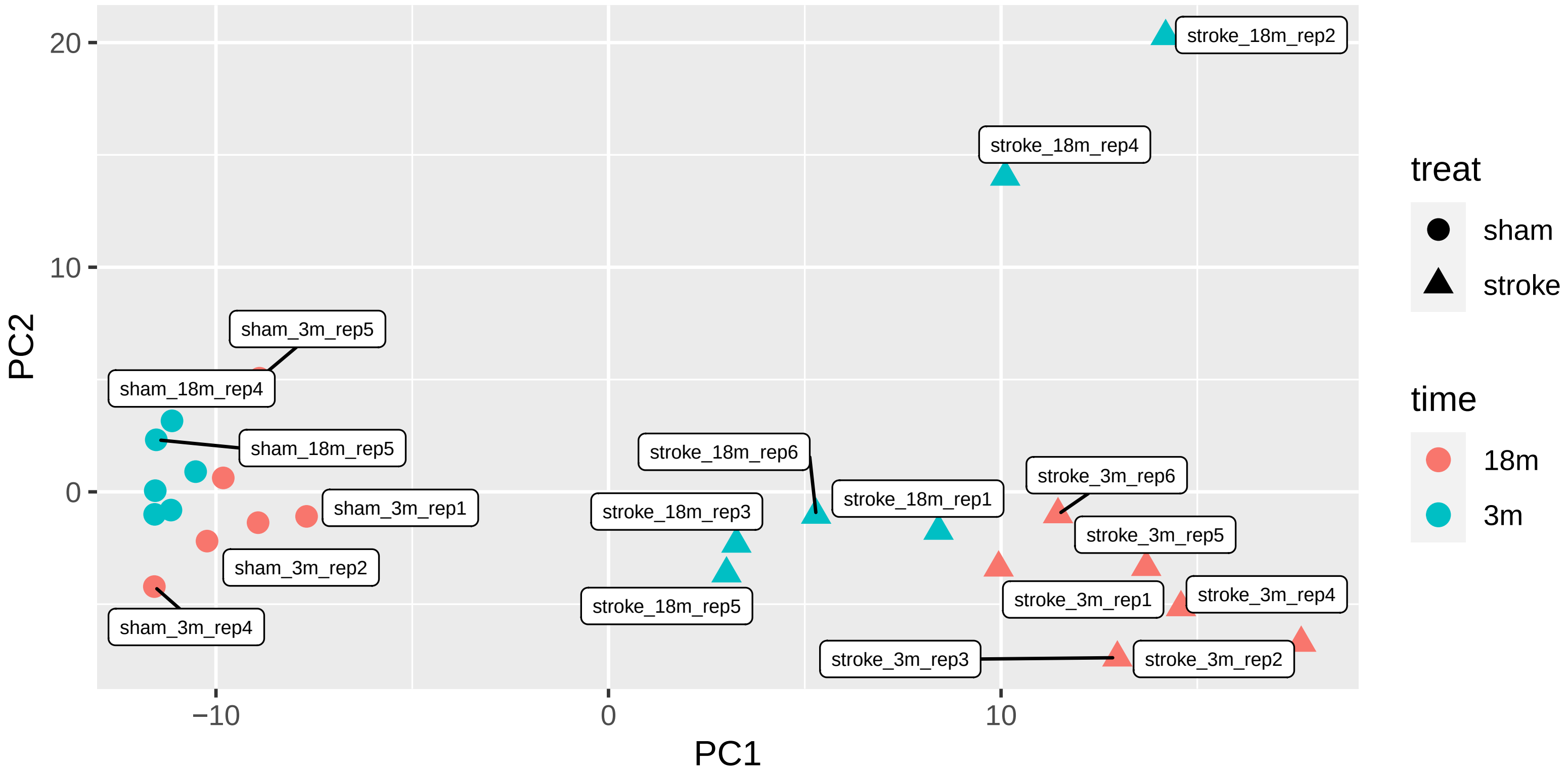

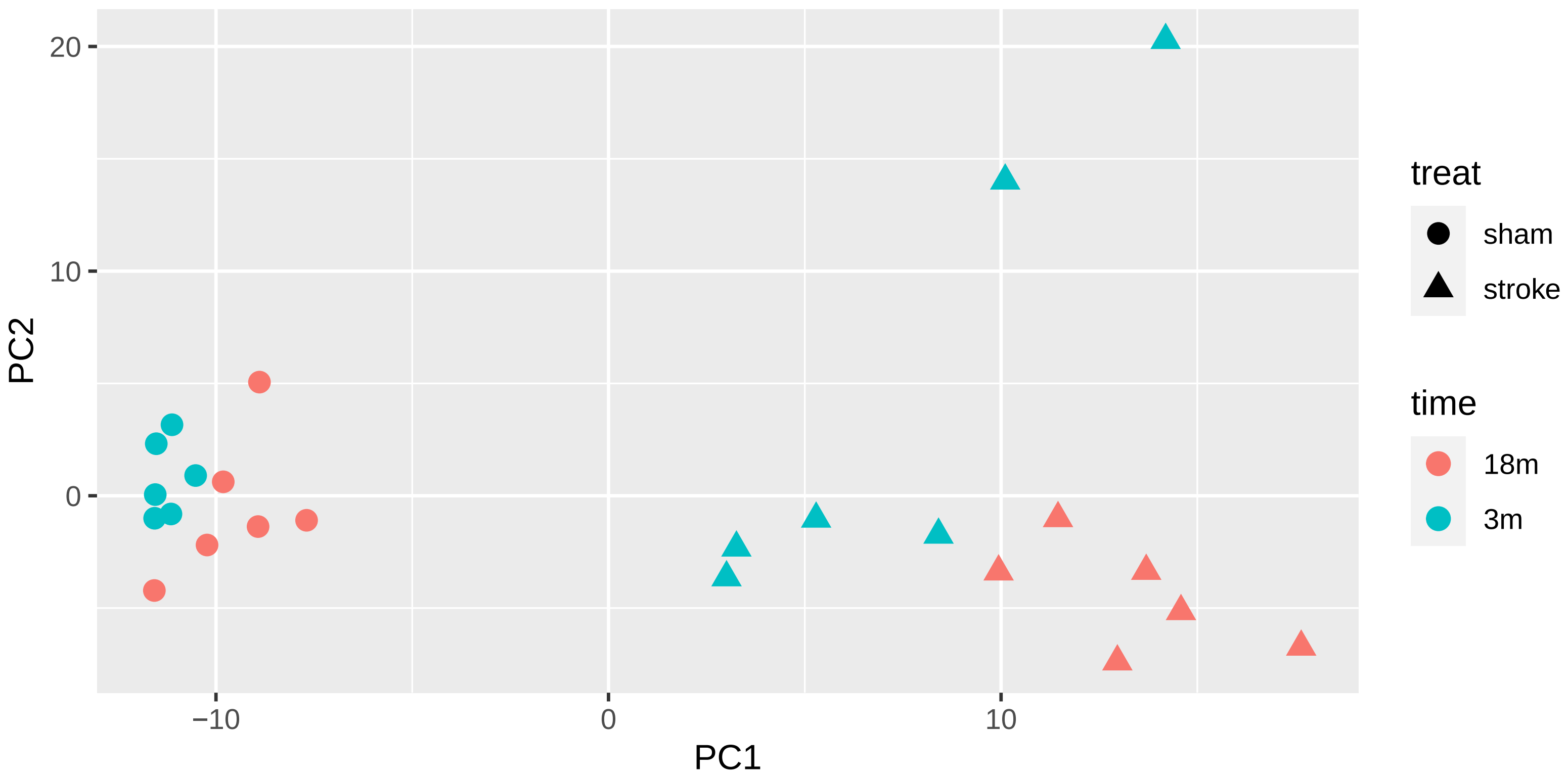

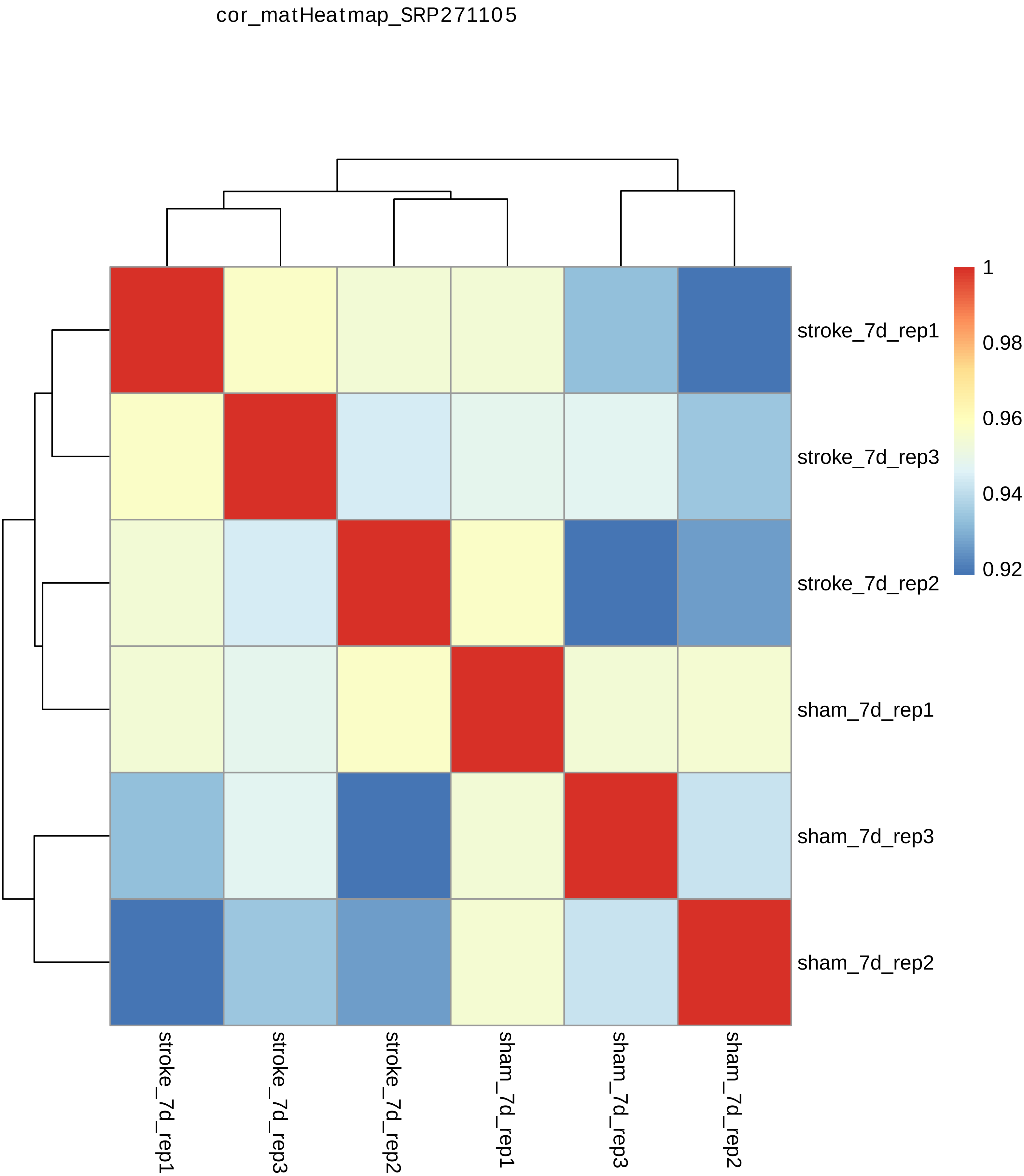

cor_matHeatmap_SRP271105
stroke_7d_rep1
stroke_7d_rep3
stroke_7d_rep2
sham_7d_rep1
sham_7d_rep3
sham_7d_rep2
1
0.98
0.96
0.94
0.92

sample_pca_SRP271105

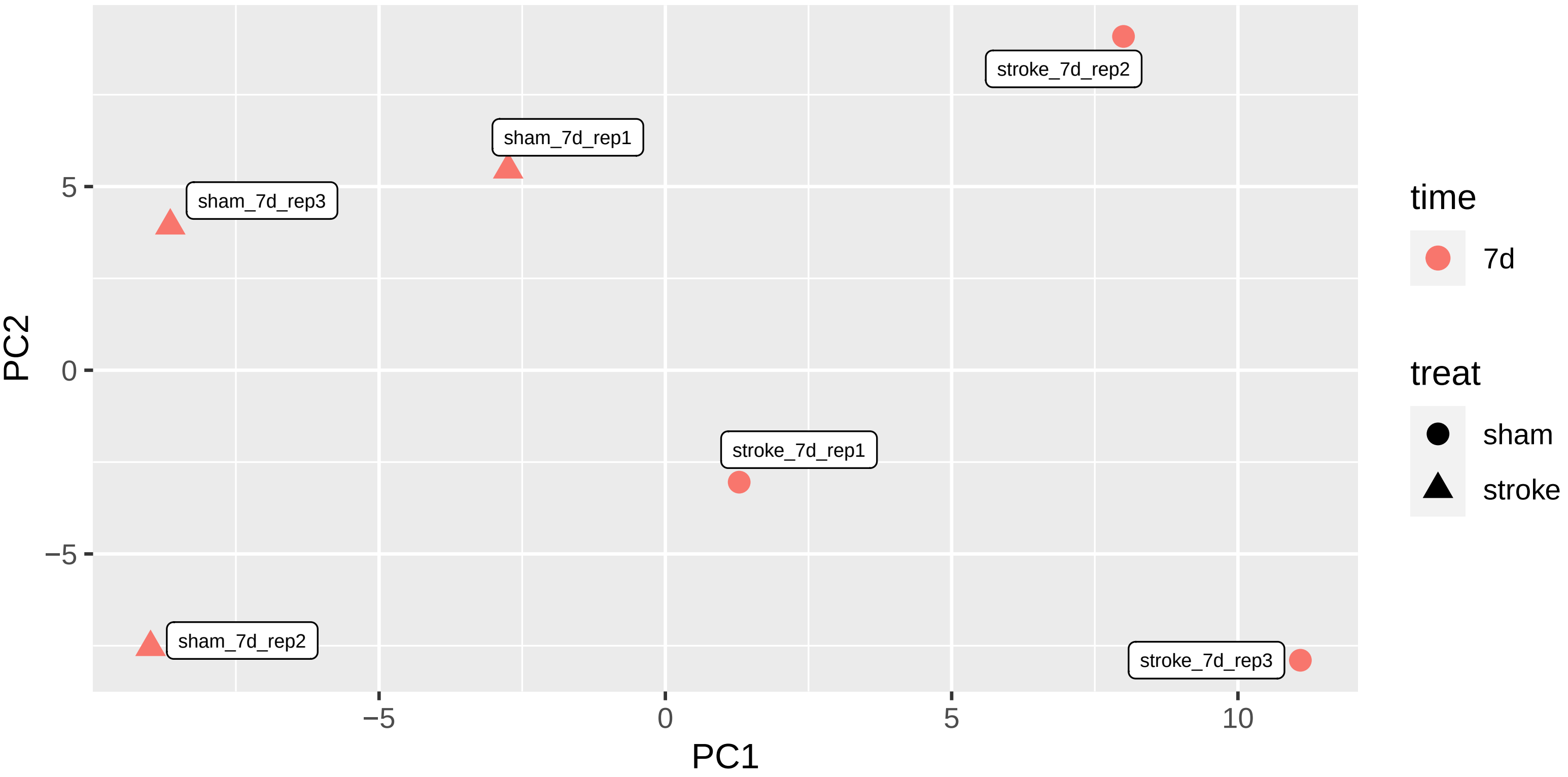

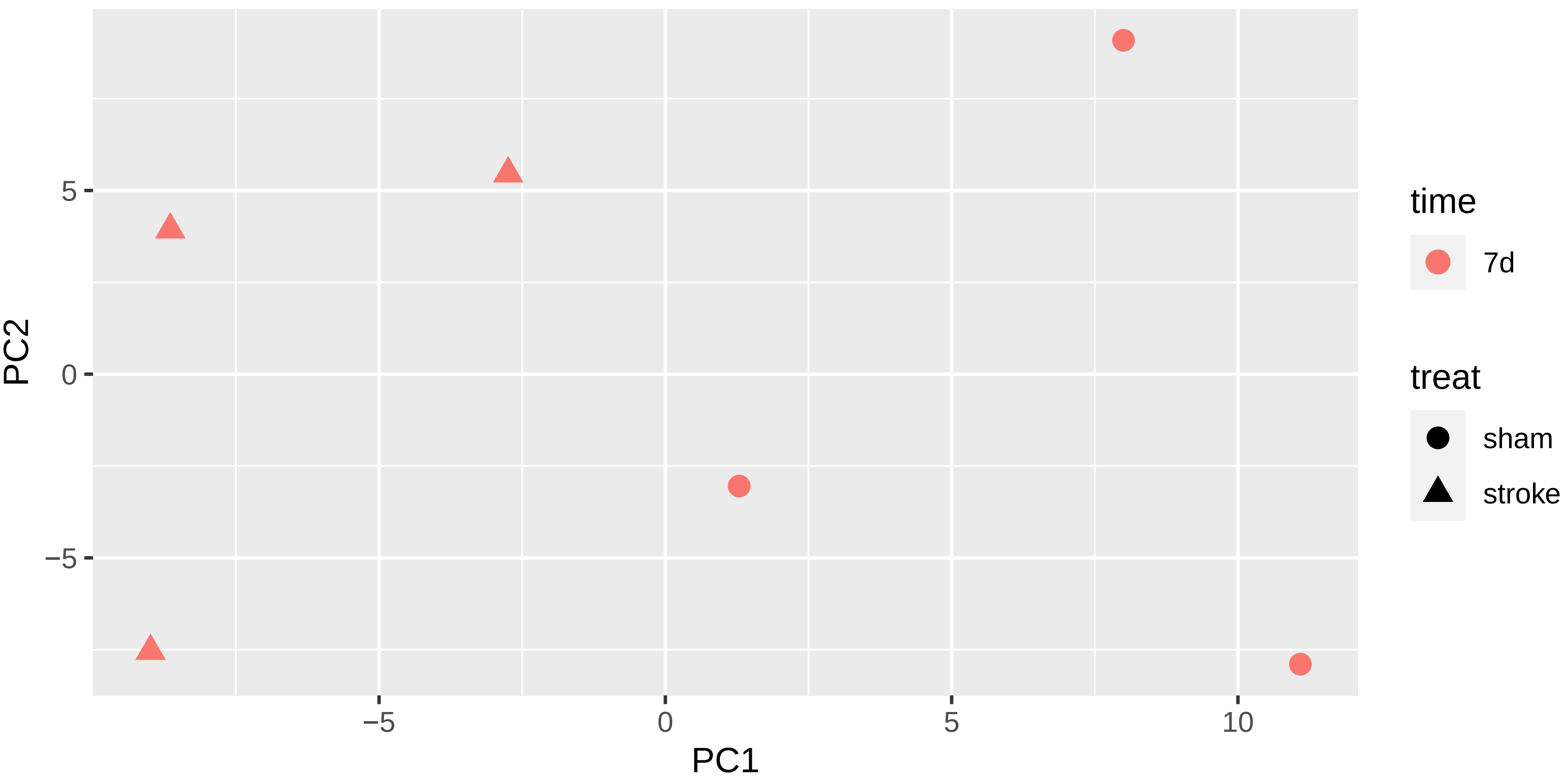

cor_matHeatmap_SRP298712

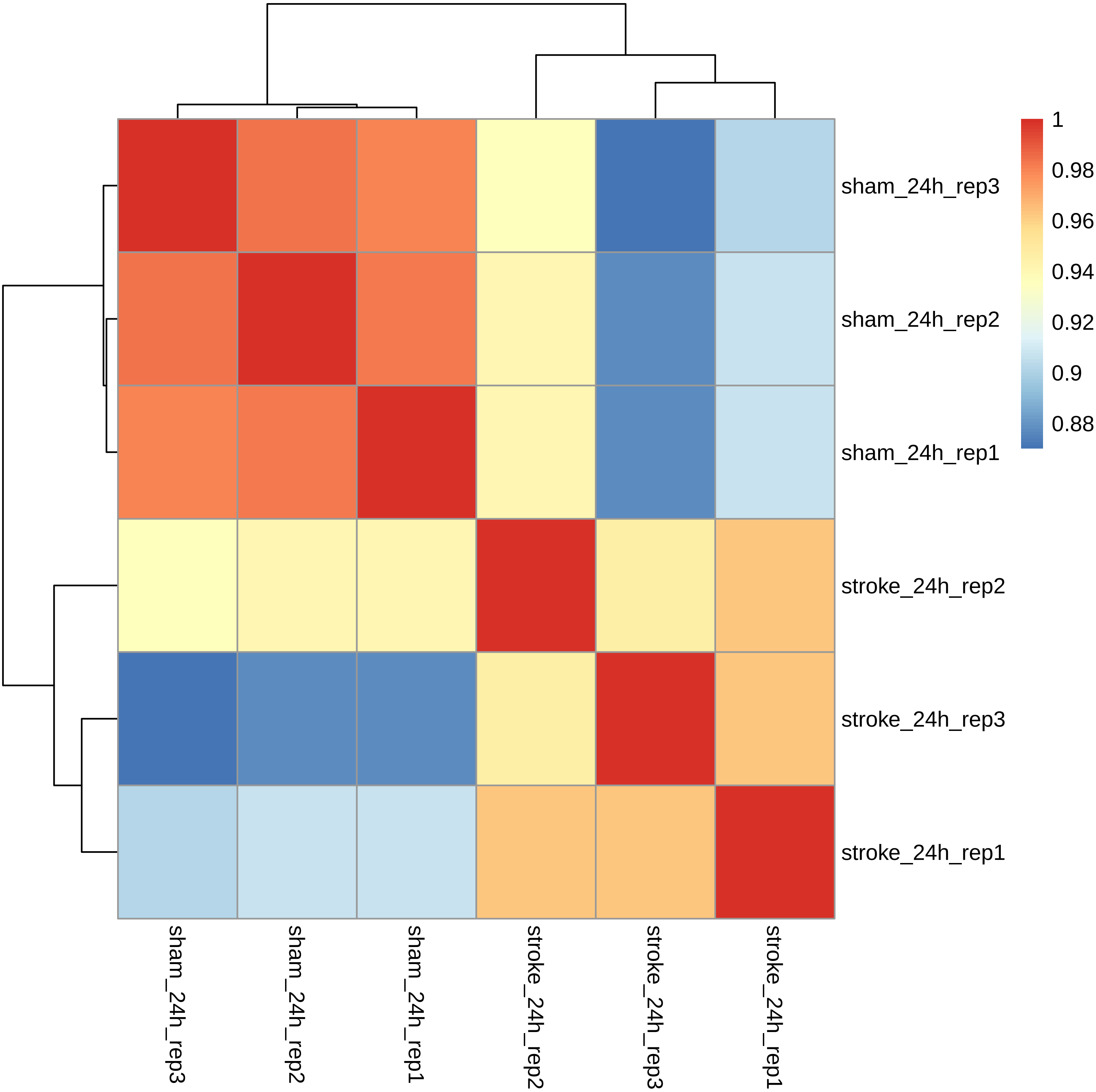

sample_pca_SRP298712

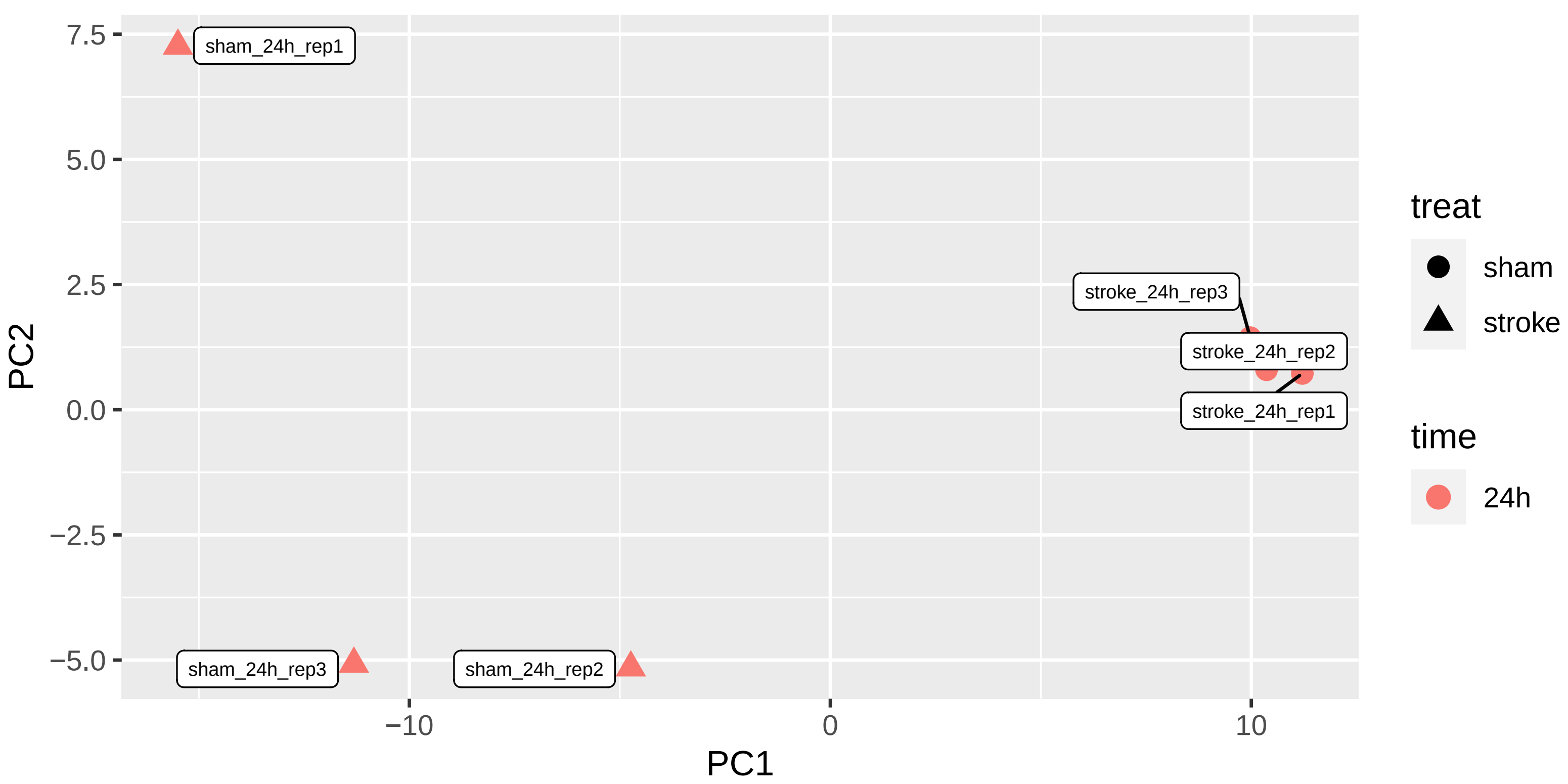

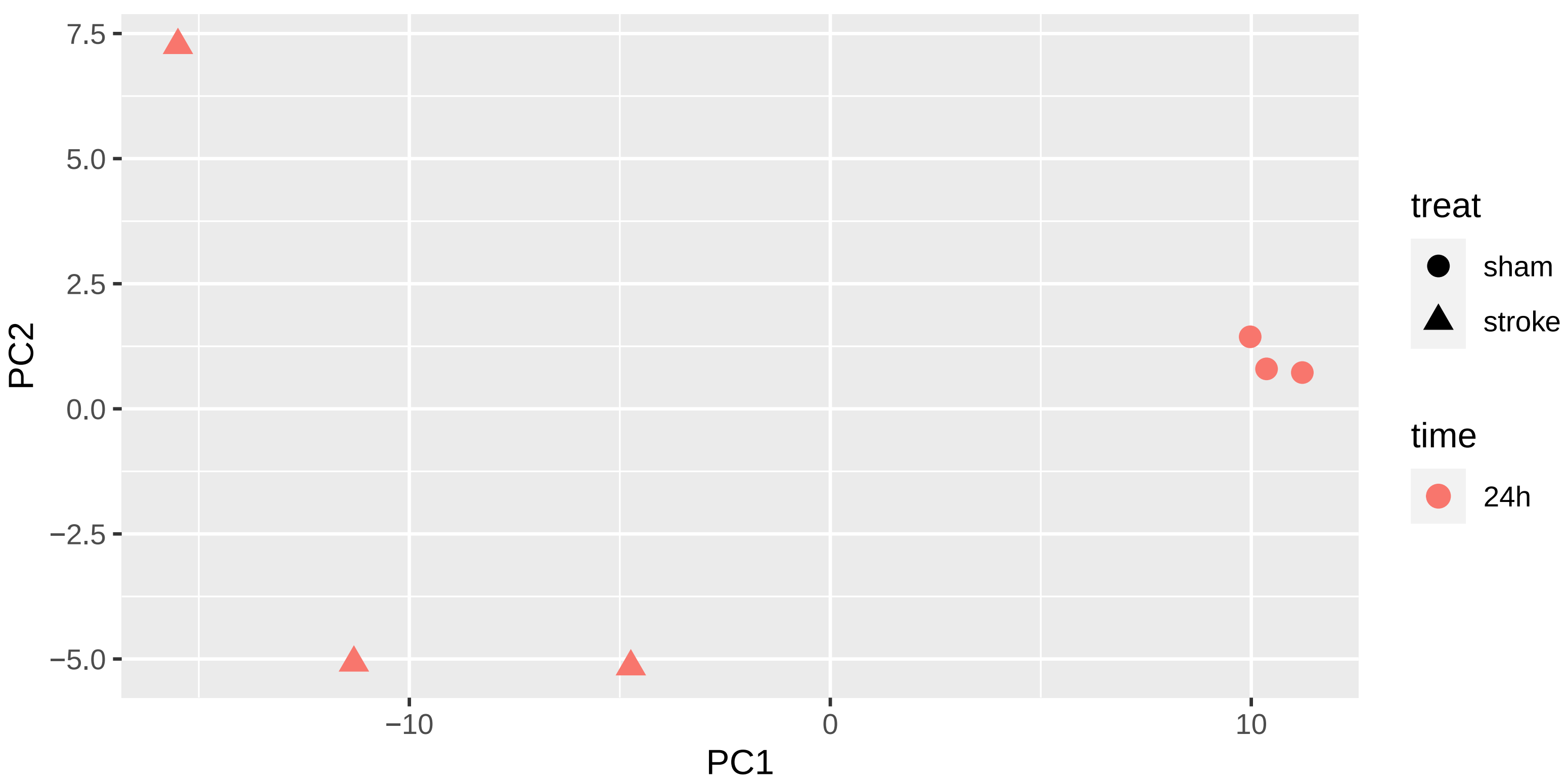

cor_matHeatmap_SRP316861

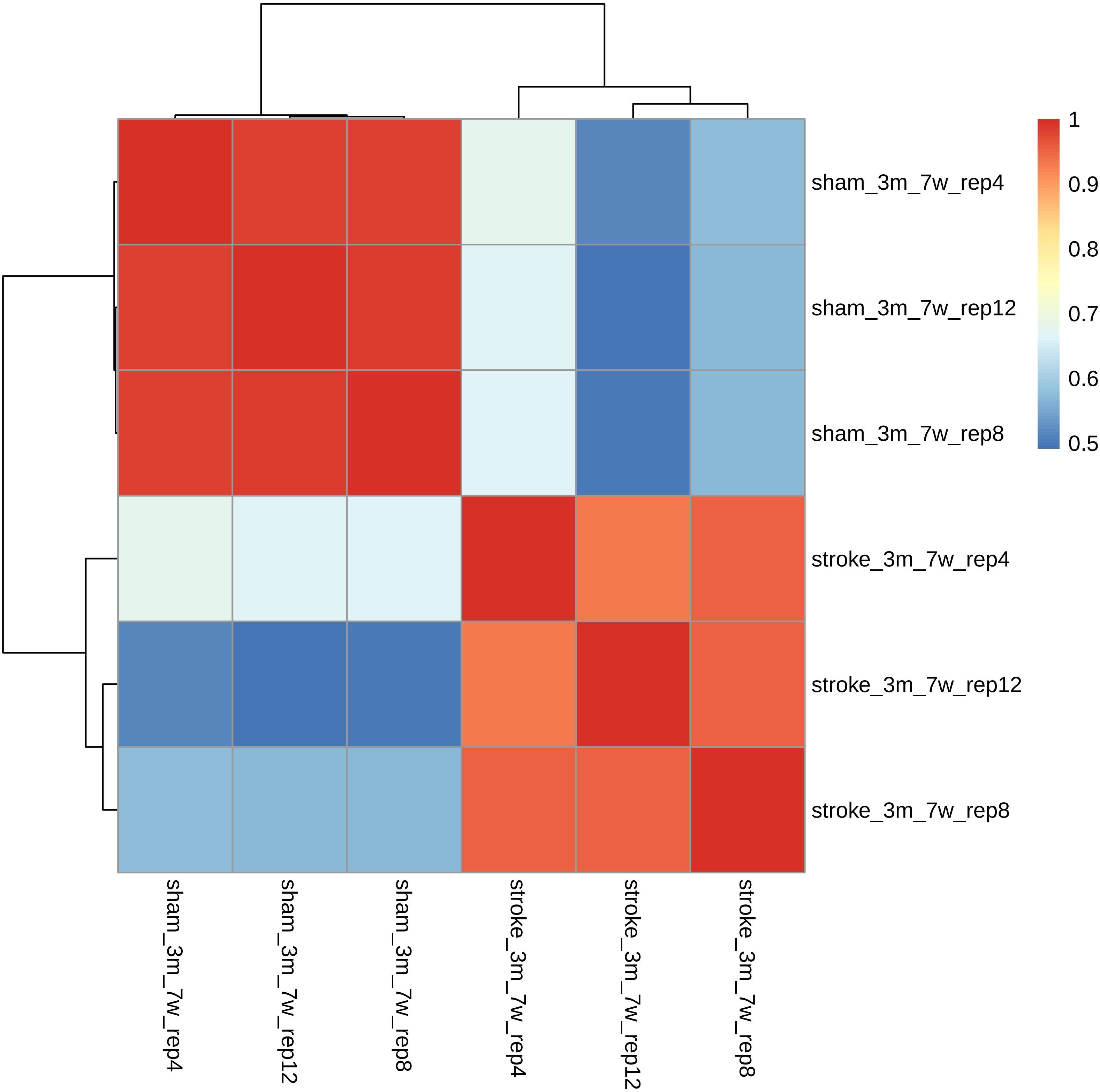

sample_pca_SRP316861

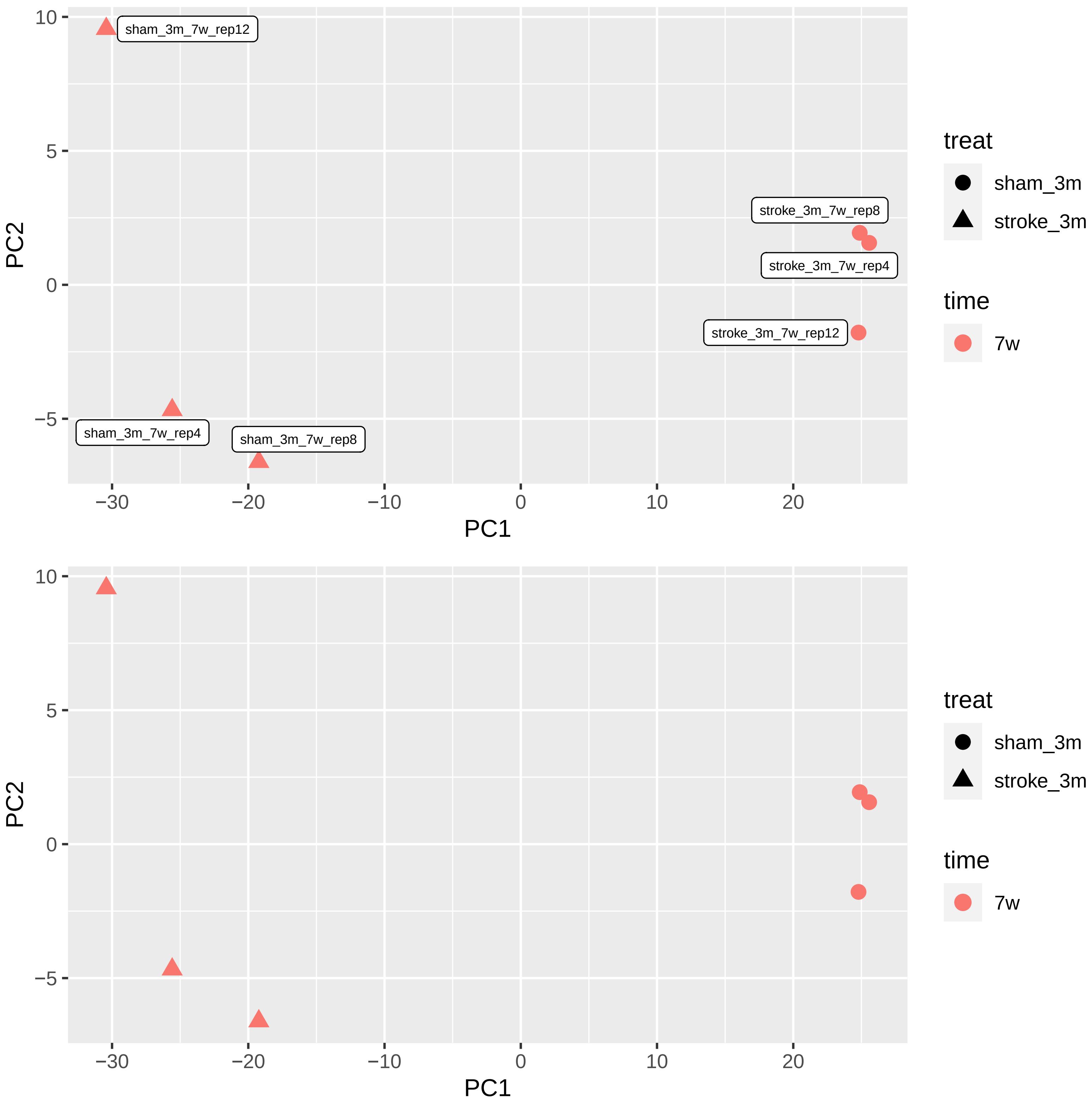

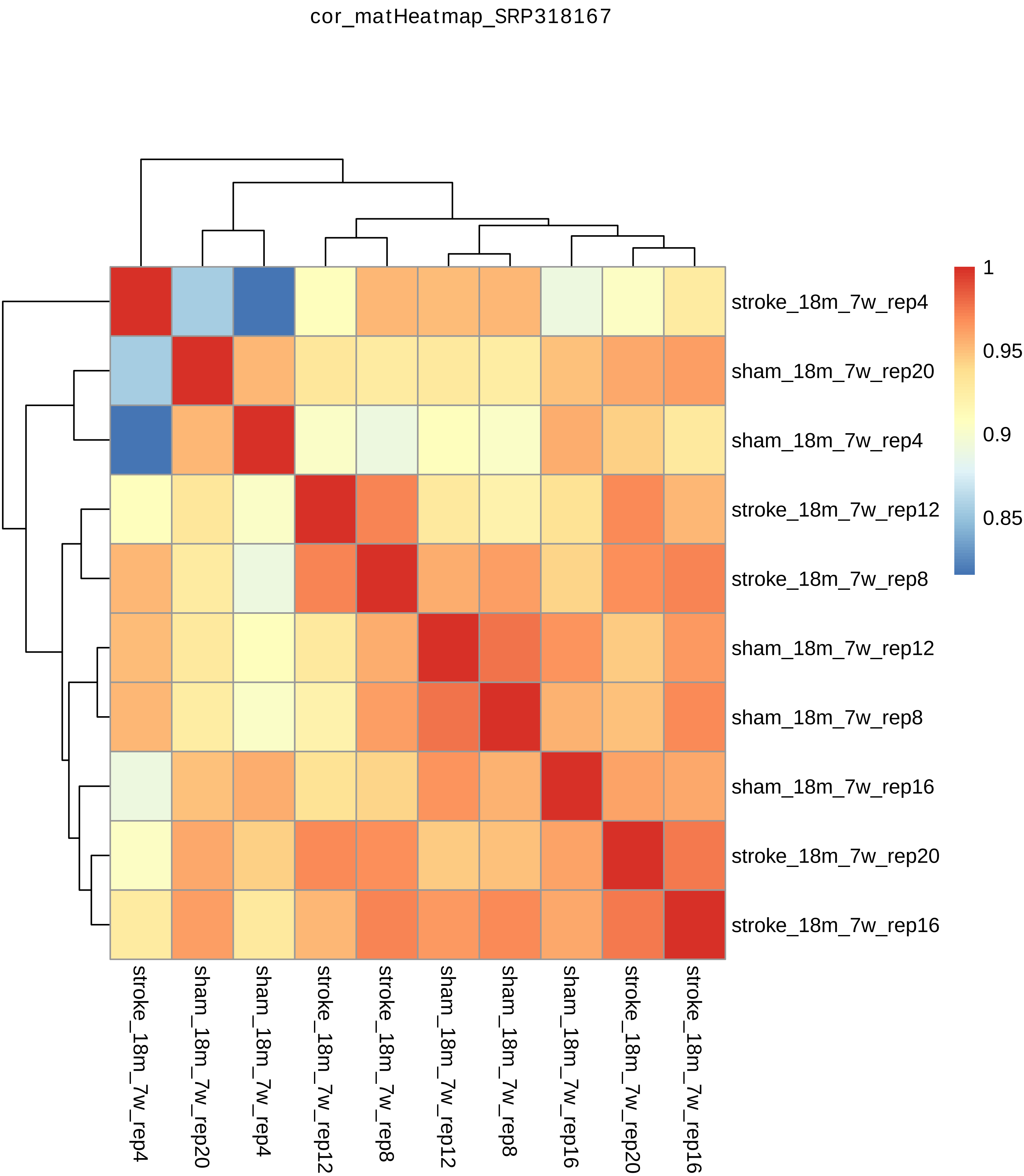
cor_matHeatmap_SRP318167
stroke_18m_7w_rep4
sham_18m_7w_rep20
sham_18m_7w_rep4
stroke_18m_7w_rep12
stroke_18m_7w_rep8
sham_18m_7w_rep12
sham_18m_7w_rep8
sham_18m_7w_rep16
stroke_18m_7w_rep20
stroke_18m_7w_rep16
1
0.95
0.9
0.85

sample_pca_SRP318167

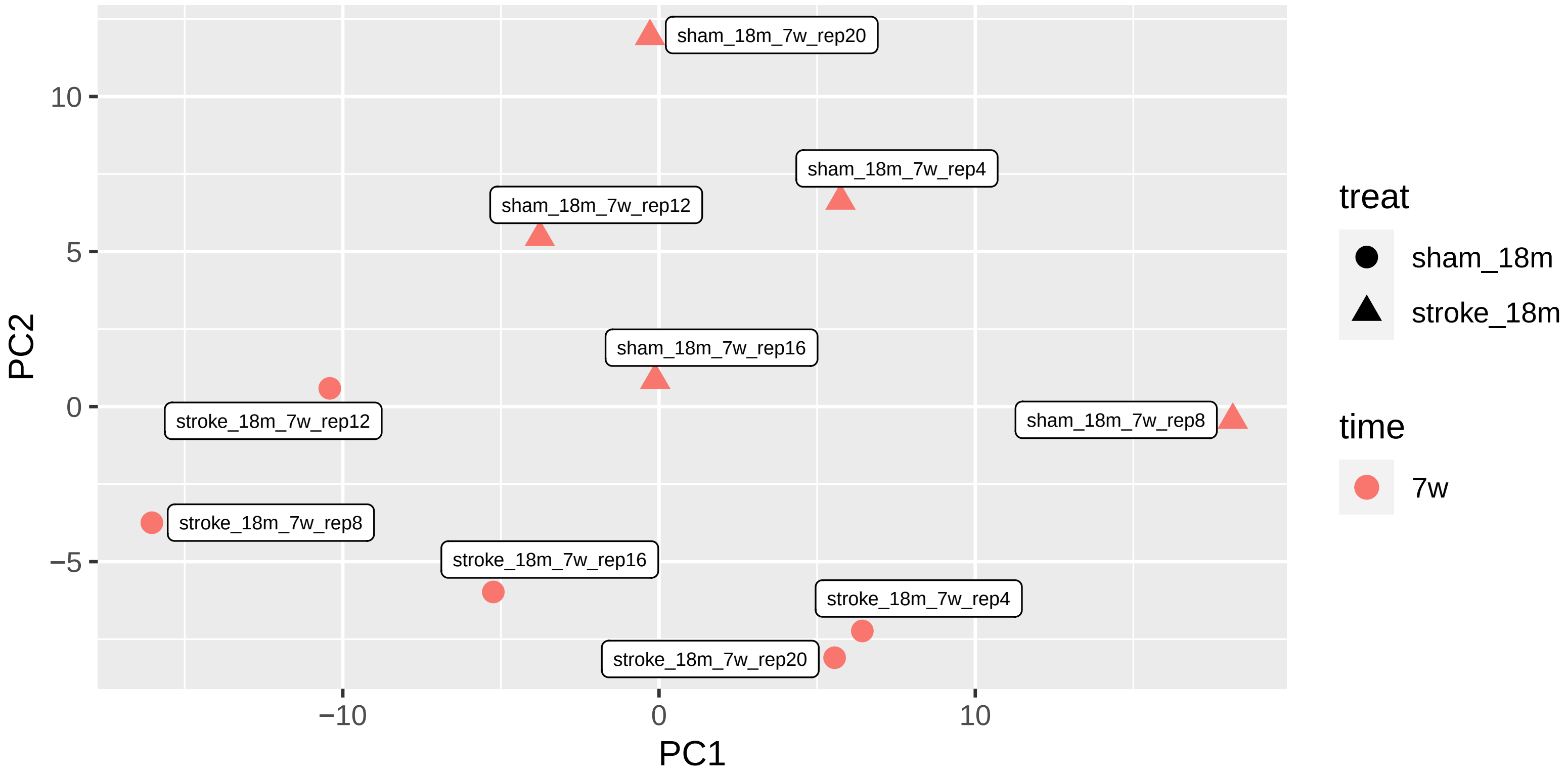

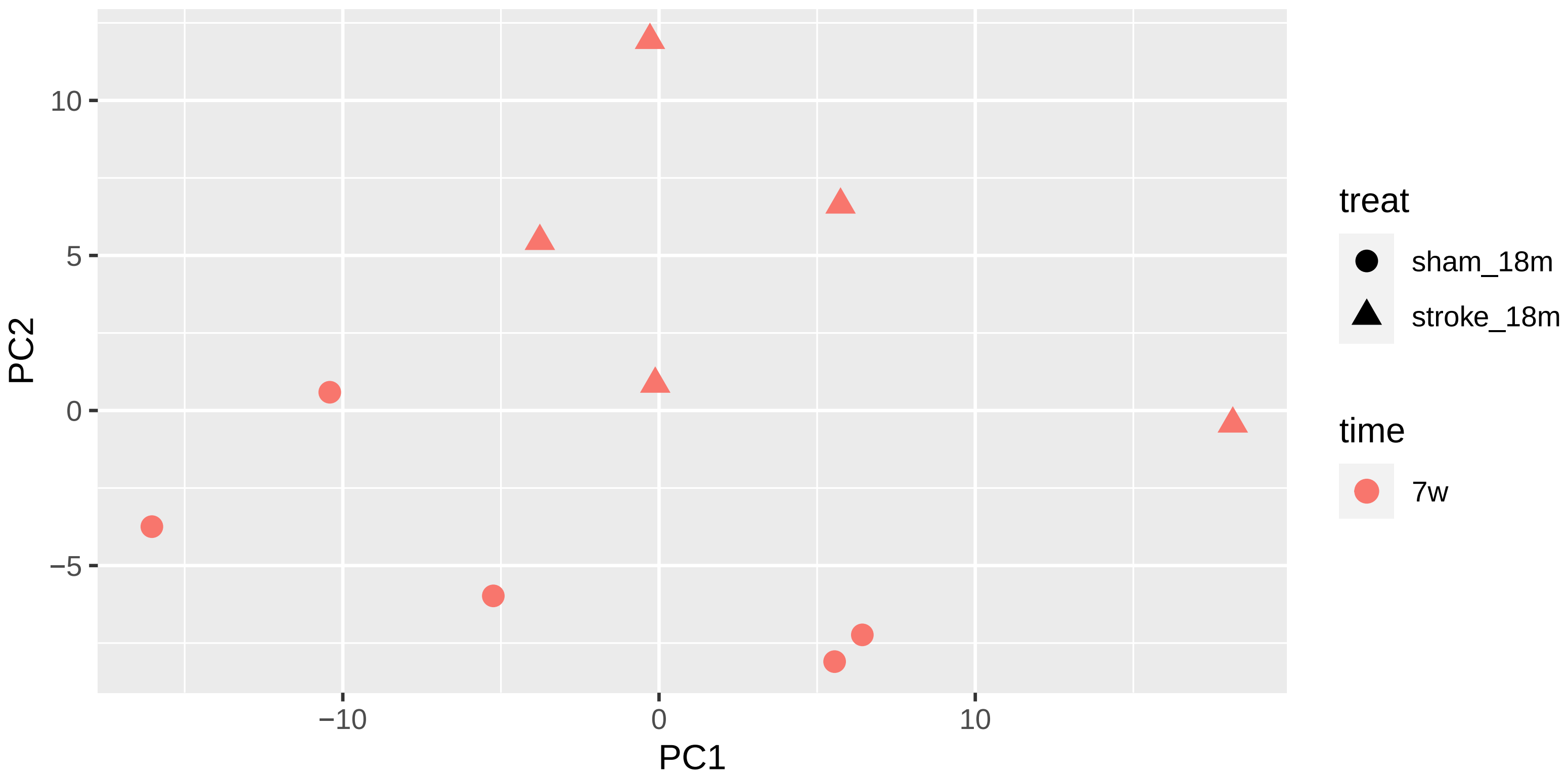

Supplement: Supplementary file 1 [file SupplementaryFile2.pdf]
