# Supplementary material for: Integrated machine learning and transcriptomics reveal immune infiltration-related orthologous transcription genes in cerebral ischemic injury
Source: Front Immunol. 2026 Aug 11;17:1700109. doi: 10.3389/fimmu.2026.1700109 (PMC13503618; doi:10.3389/fimmu.2026.1700109)
Supplement: Supplementary file 2 [file SupplementaryFile1.docx]

### Supplementary figures

Fig. S1

Fig. S1 Merging datasets and removing batch effects. **(A)** Box plot showing the sample distribution of each data set after batch correction. **(B)** UMAP analysis showing the sample distribution of each dataset after batch correction. **(C)** PCA results of the datasets after batch correction. UMAP, Uniform Manifold Approximation and Projection; PCA, Principal Component Analysis.

Fig. S2

Fig. S2 Heatmap of stably up- and down-regulated DEGs in rodents. **(A, B)** Heatmap of robustly up- and down-regulated DEGs in mice. **(C, D)** Heatmap of robustly up- and down-regulated DEGs in rat models. DEGs, differentially expressed genes.

Fig. S3

Fig. S3 Harmonization of the data. **(A)** Box plots showing the sample distribution of each dataset after batch effect removal in the in-house dataset. **(B)** PCA results after batch correction in the in-house dataset. PCA, Principal Component Analysis.

Fig. S4

Fig. S4 Analysis of immune infiltration for key orthologous TFs in mouse public datasets. **(A)** Stacked bar chart showing the distribution of immune cells in the Sham and Ischemia groups. **(B)** Comparison of immune cell infiltration levels in the Sham and Ischemia groups from the public dataset. **(C)** Heatmap displays immune cell expressions in different groups across the two datasets. **(D)** Heatmap illustrating correlations among immune cells in the public dataset. **(E)** Analysis of the correlation between core TFs and various immune cell types. **(F)** Correlation analysis of expression levels of 5 key TFs with M1 and M2 macrophage markers. TFs, Transcription factors. * *P*<0.05, *** *P* < 0.001.

Fig. S5

Fig. S5 Immune infiltration analysis of core orthologous TFs in public rat datasets. **(A)** Stacked bar chart showing the distribution of immune cells in the Sham and Ischemia groups. **(B)** Comparison of immune cell infiltration between Sham and Ischemia groups using the SRP148632 dataset. **(C)** Heatmap showing correlations among immune cells. **(D)** Heatmap depicting correlations between immune cells within the SRP148632 dataset. **(E)** Correlation of core TFs with immune cell types. **(F)** Analysis of correlations between the expression of 5 hub TFs and M1/M2 macrophage markers. TFs, Transcription factors. * *P*<0.05.

Fig. S6

Fig. S6 Potential relationship between core TFs and microglia. **(A, B)** FPKM values of microglial markers Tmem119 and Trem2 in our mouse MCAO and rat HIE datasets**.** The 95% CIs for the between‑group differences are as follows: in the mouse MCAO model, Tmem119: –3.904 to 0.8903, Trem2: 0.5857 to 5.669; in the rat HIE model, Tmem119: –0.8859 to 1.474, Trem2: –0.5955 to 21.83. **(C, D)** Heatmap showing correlations between key TFs (*Atf3*, Maff, Cebpa, Relb, Myc) and microglial markers (Tmem119, Trem2) in public datasets. TFs, Transcription factors; FPKM, Fragments per kilobase of exon model per million mapped fragments; MCAO, Middle Cerebral Artery Occlusion; HIE, Hypoxic-ischemic encephalopathy. * *P*<0.05.
