# Supplementary material for: Integrated machine learning and transcriptomics reveal immune infiltration-related orthologous transcription genes in cerebral ischemic injury
Source: Front Immunol. 2026 Aug 11;17:1700109. doi: 10.3389/fimmu.2026.1700109 (PMC13503618; doi:10.3389/fimmu.2026.1700109)
Supplement: Supplementary file 3 [file Table2.docx]

**Supplementary Table 2. Antibodies in this study**

| Antibodies | Vendor | Catalogue  Number | Host | Dilution Factor |
| --- | --- | --- | --- | --- |
| C/EBP alpha  ATF3  RelB  IBA1  Anti-mouse IgG (H+L), F(ab')2 Fragment (Alexa Fluor® 488 Conjugate)  Anti-rabbit IgG (H+L), F(ab')2 Fragment (Alexa Fluor® 555 Conjugate)  FITC conjugated Goat Anti-Rabbit IgG (H+L)  Alexa Fluor® 594-conjugated Goat Anti-Mouse IgG (H+L) | Affinity  Affinity  Servicebio  Servicebio  CST  CST  Servicebio  Servicebio | #AF6333  #DF3110  GB11353  GB12105  4408S  4413S  GB22303  GB28303 | Rat  Rat  Rat  Mouse  Goat  Goat  Goat  Goat | 1:100  1:100  1:100  1: 500  1:2000  1:2000  1:200  1:200 |

**Supplementary Table 3. Primers for qRT-PCR**

| Genes | Primer sequence | Product  (bp) |
| --- | --- | --- |
| Gapdh-S  Gapdh-A  Atf3-S  Atf3-A  Maff-S  Maff-A  Cebpa-S  Cebpa-A  Relb-S  Relb-A  Myc-S  Myc-A  Aif1-S  Aif1-A  Tmem119-S  Tmem119-A  Trem2-S  Trem2-A | CTGGAGAAACCTGCCAAGTATG  GGTGGAAGAATGGGAGTTGCT  GGAGTCAGTCACCATCAACAACAG  GCCTTCAGTTCGGCATTCA  ACCTTCTGCGAAAATGTCCGT  ATCAGCGCCTCATCCGACA  GCCAAGAAGTCGGTGGATAAGA  GGCGGTCATTGTCACTGGTC  TAACAACTTGGGCATCCAGTGT  GTTCTTCAGGGAGCCAGCATT  TGGAGGAGACATGGTGAATCAG  AAGCCGCTCCACATACAGTCC  GCTCCGAGGAGACGTTCAGTTAC  GTTGGCTTCTGGTGTTCTTTGTTT  GCCTACTACCCGTCCTCTTTCC  CGACCCACCCTTCACCTGTT  CCAAGCCCTCAACACCACA  ACCGTGCTCCCATTCTGCTT | 138  204  102  151  106  96  118  237  201 |
